# Large-area, self-healing block copolymer membranes for energy conversion

**DOI:** 10.1038/s41586-024-07481-2

**Published:** 2024-06-05

**Authors:** Christian C. M. Sproncken, Peng Liu, Justin Monney, William S. Fall, Carolina Pierucci, Philip B. V. Scholten, Brian Van Bueren, Marcos Penedo, Georg Ernest Fantner, Henricus H. Wensink, Ullrich Steiner, Christoph Weder, Nico Bruns, Michael Mayer, Alessandro Ianiro

**Affiliations:** 1 Adolphe Merkle Institute, University of Fribourg, Fribourg, Switzerland; 2 Swiss National Center for Competence in Research (NCCR) Bio-inspired Materials, University of Fribourg, Fribourg, Switzerland; 3 Department of Materials, ETH Zürich, Zürich, Switzerland; 4 Laboratoire de Physique des Solides - UMR 8502, CNRS, Université Paris-Saclay, Orsay, France; 5 Laboratory for Bio- and Nano-Instrumentation, Institute of Bioengineering, School of Engineering, EPFL, Lausanne, Switzerland; 6 Department of Pure and Applied Chemistry, University of Strathclyde, Glasgow, UK; 7 Department of Chemistry and Centre for Synthetic Biology, Technical University of Darmstadt, Darmstadt, Germany

**Keywords:** Molecular self-assembly, Molecular self-assembly

## Abstract

Membranes are widely used for separation processes in applications such as water desalination, batteries and dialysis, and are crucial in key sectors of our economy and society^1^. The majority of technologically exploited membranes are based on solid polymers and function as passive barriers, whose transport characteristics are governed by their chemical composition and nanostructure. Although such membranes are ubiquitous, it has proved challenging to maximize selectivity and permeability independently, leading to trade-offs between these pertinent characteristics^2^. Self-assembled biological membranes, in which barrier and transport functions are decoupled^3,4^, provide the inspiration to address this problem^5,6^. Here we introduce a self-assembly strategy that uses the interface of an aqueous two-phase system to template and stabilize molecularly thin (approximately 35 nm) biomimetic block copolymer bilayers of scalable area that can exceed 10 cm^2^ without defects. These membranes are self-healing, and their barrier function against the passage of ions (specific resistance of approximately 1 MΩ cm^2^) approaches that of phospholipid membranes. The fluidity of these membranes enables straightforward functionalization with molecular carriers that shuttle potassium ions down a concentration gradient with exquisite selectivity over sodium ions. This ion selectivity enables the generation of electric power from equimolar solutions of NaCl and KCl in devices that mimic the electric organ of electric rays.

## Abstract (web summary)

We introduce a self-assembly strategy that uses the interface of an aqueous two-phase system to template and stabilize molecularly thin biomimetic block copolymer bilayers of scalable area that can exceed 10 cm^2^ without defects.

## Main

Cell membranes regulate the exchange of matter within and across living cells with excellent efficiency and selectivity^3,4^. Their barrier function is provided by a self-assembled phospholipid bilayer which, in spite of its molecular thickness, is virtually impermeable to most hydrophilic and charged molecules. Extreme selectivity is achieved through proteins that are embedded in the bilayer and operate as diffusion-controlled carriers, channels or chemically fuelled pumps^3^. The molecular-scale thickness of biological membranes is key to high transport rates for desired species, and the dynamic nature of such supramolecular assemblies allows them to morph their shape and self-heal without losing functionality^4^. Mimicking the intricate design of cell membranes with lipids or amphiphilic block copolymers (BCPs) has thus emerged as a promising approach to developing high-performance artificial membranes^7,8^. However, the fabrication of macroscopic membranes via molecular self-assembly requires control over the assembly process across length scales that span at least 14 orders of magnitude—that is, from the molecular level to a surface area of several square centimetres and beyond. To date, this scale has not been achieved by molecular design with conventional self-assembly strategies. Phospholipids, the molecular building blocks of natural membranes, have evolved to function best at the cellular-length scale but their assemblies become increasingly unstable when their area exceeds 1 mm^2^ (refs. ^9,10^). Amphiphilic BCPs represent a versatile synthetic alternative to natural phospholipids because they exhibit similar self-assembly behaviour but form membranes with mechanical properties superior to those of phospholipids^8,11^. Nevertheless, without a solid support^8,12,13^, the maximum achievable area of free-standing BCP membranes is also limited to the square-millimetre range^14–16^, which is too small for practical separation or energy conversion applications.

## The solvent-displacement method

**Fig. 1:**
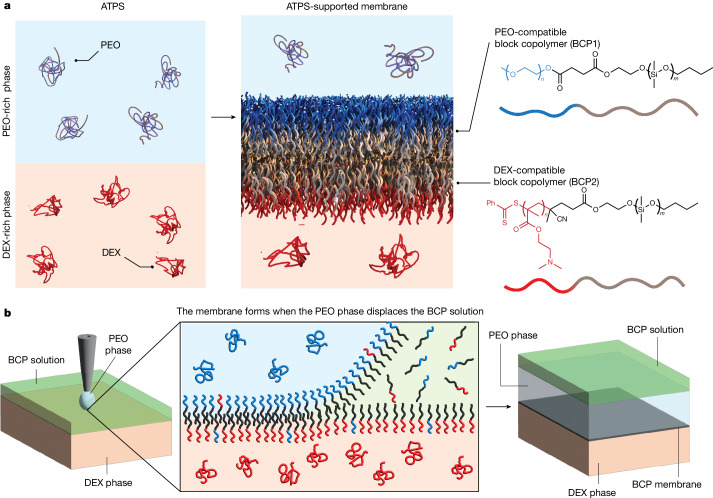
Self-assembly of BCP bilayers supported by an ATPS. **a**, Schematic representation of an ATPS based on aqueous solutions of PEO and DEX (left), an ATPS-supported self-assembled bilayer (middle) and the two BCPs used to form the bilayer (right). **b**, Schematic depiction of the solvent-displacement method introduced here to form large-area membranes. The green phase represents a BCP solution in a water-immiscible organic solvent (typically toluene). Because the density of the aqueous PEO phase is lower than that of the aqueous DEX phase but higher than that of the solution of BCPs in toluene, sedimentation of the PEO phase at the interface displaces the BCP solution, which rises to the top of the vessel.

Like many amphiphilic molecules, BCPs accumulate at interfaces because this process minimizes the interfacial energy of the system. We harnessed this thermodynamic driving force to control BCP self-assembly in two dimensions and stabilized the resulting membranes by exploiting the interface between an aqueous two-phase system (ATPS; Fig. 1a), which forms when immiscible aqueous solutions of chemically incompatible polymers are brought into contact^17,18^.

The interfacial tension (*γ*) of ATPSs is very small; for the poly(ethylene oxide) (PEO) and dextran (DEX) systems investigated here, *γ* ≲ 0.1 mJ m^−2^ (0.02 *k*_B_*T* nm^−2^ where *k*_B_ is the Boltzmann constant and *T* is temperature), which is two to three orders of magnitude lower than the interfacial tension between water and air or water and alkanes^19^. On the molecular scale, such a small interfacial tension does not provide sufficient driving force for interfacial assembly. However, as we demonstrate here, it can provide a substantial stabilizing contribution once the membrane is formed (see Supplementary Discussion 1 for thermodynamic details).

As shown in Fig. 1, we used two different BCP types with the same hydrophobic but different hydrophilic blocks that extend into the PEO (BCP1) and DEX (BCP2) phases of the ATPS, respectively, so that an asymmetric bilayer can form at the interface. The composition of BCP1 and BCP2 is presented in Fig. 1a and discussed in Methods. We successfully tested also other types of BCP (Methods, Supplementary Methods and Supplementary Discussions).

**Fig. 2:**
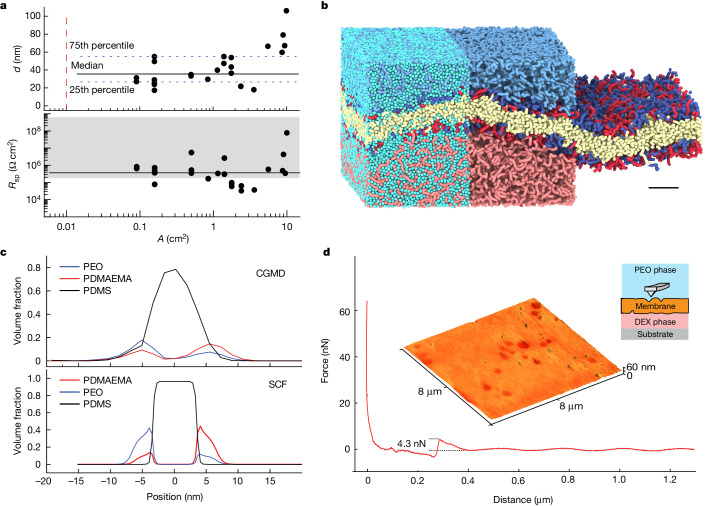
Characterization of ATPS-supported membranes composed of BCP1 and BCP2. **a**, Membrane thickness (*d*) determined from capacitive measurements and specific electric resistance (*R*_sp_) of membranes of varying surface area (*A*); black dots represent single membranes. Top, the black horizontal line is the median *d* value and the blue dotted lines indicate the first and third quartiles; the red dashed vertical line indicates the area of the largest self-assembled BCP membrane reported in the literature^16^; bottom, the grey region indicates the reference *R*_sp_ range for pure and oil-swollen lipid bilayers^38,39^. **b**, Structure of an ATPS-supported BCP membrane resulting from coarse-grained molecular dynamics (CGMD) simulations. The CGMD snapshot is divided into three segments. The left segment shows the full composition of the ATPS interface, with water (cyan), PEO homopolymers (light blue), DEX (light red), PEO blocks (BCP1, dark blue), poly[2-(dimethylamino) ethyl methacrylate] (PDMAEMA) blocks (BCP2, dark red) and poly(dimethyl siloxane) (PDMS, cream). The central segment shows only the BCPs and homopolymers and the right segment shows only the ATPS-supported bilayer. Scale bar, 10 nm. **c**, Concentration profiles illustrating the composition of the bilayer in the direction perpendicular to the membrane plane, obtained via CGMD simulations (top) and self-consistent field (SCF) computations (bottom). **d**, AFM characterization of ATPS-supported membranes in liquid (inset). The red line represents the cantilever force versus tip-sample distance when the cantilever is moved towards the sample. The abrupt change on the force curve at 0.3 µm distance represents the penetration of the membrane, from which we obtained a penetration force of 4.3 nN. The darker regions on the membrane plane represent small variations in membrane thickness of approximately 6 nm (Supplementary Fig. 1), and hence are much smaller than the total membrane thickness (roughly 40 nm for this membrane) determined via capacitive measurements. Source Data

Because the low interfacial tension of the ATPS does not provide a sufficient driving force at the molecular scale to guide interfacial assembly, we devised a two-step templating strategy based on the displacement of a water-immiscible organic solvent from the interface of the two aqueous phases (Fig. 1b). We added the DEX solution, which is denser than the PEO solution, to a vessel whose cross-section defines the area of the membrane. Then, we gently placed a solution of the two BCPs in a suitable organic solvent, such as toluene, on top of the DEX phase (Fig. 1b). We chose these conditions because they favour the formation of a BCP monolayer at the interface between the aqueous and organic phases^20^. The monolayer is likely richer in BCP2 than BCP1, as indicated by fluorescence resonance energy transfer measurements and computer simulations of the bilayers (Supplementary Discussions 3–5 and Fig. 2), because interactions between the DEX solution and the PEO block of BCP1 are enthalpically unfavourable. Finally we slowly added the aqueous PEO solution from the top. This phase is denser than the organic solvent and sinks towards the DEX phase, thereby displacing the organic solvent which rises to the top. During this displacement process, a second monolayer, richer in BCP1 than BCP2 (Supplementary Discussions 3–5 and Fig. 2c), forms at the interface between the organic phase and the PEO solution. When the PEO and DEX phases meet, the two monolayers merge into a planar BCP bilayer (Fig. 1b).

The solvent-displacement method reported here has elements in common with the droplet interface bilayer method^21^ in which two aqueous droplets, coated by lipid monolayers following immersion in an oil–lipid mixture, are brought into contact to form a bilayer. The solvent-displacement method, however, introduces the concept of forming and stabilizing macroscopic self-assembled membranes by minimizing the interfacial free energy of an ATPS. This concept is realized by selection of BCPs with hydrophilic blocks that are compatible with the aqueous polymer phases on either side of the membrane. Control experiments that attempted to implement the solvent-displacement method using two PEO solutions as phases 1 and 2 together with BCP1, or two DEX solutions together with BCP2, did not result in the formation of stable membranes, confirming the essential stabilizing role of the ATPS interface (Supplementary Discussion 2).

We applied the solvent-displacement method using various water-immiscible organic solvents (Supplementary Discussion 6). Of the six solvents we tested, those with viscosity *η* > 0.5 mPa s such as toluene yielded stable membranes. Supplementary Discussion 6 provides an interpretation of this unexpected dependence on solvent viscosity. We selected toluene for most of the subsequent studies because it is commonly available and broadly used in polymer science. The water–toluene interface is sharp and characterized by an interfacial tension of *γ* ≅ 35 mJ m^−2^ (8 *k*_B_*T* nm^−2^)^22^, which provides a strong driving force for the assembly of monolayers.

## BCP membranes at the water–water interface

The solvent-displacement method introduced here makes it possible to form ATPS-stabilized membranes of surface area of at least 10 cm^2^ (Fig. 2a), a size limit dictated by our experimental set-up rather than the method itself (we have not attempted to make membranes of area larger than 10 cm^2^). These membranes are three orders of magnitude larger than the largest bilayers produced with the folding method^16^ and, to our knowledge, the largest free-standing, self-assembled bilayers reported to date. Without a marked dependence on surface area, the ATPS-stabilized membranes showed a median thickness of *d* ≅ 35 nm (Fig. 2a), a specific electrical resistance exceeding 0.1 MΩ cm^2^ (similar in most cases to lipid bilayers; Fig. 2a) and stability lasting several hours (sometimes exceeding 12 h) before spontaneously breaking. We attribute the wide variability in membrane thickness to the entrapment of solvent within the bilayer, which is often observed in lipid membranes^23,24^. The variation in specific resistance is also consistent with that of oil-swollen lipid bilayers (Fig. 1a) and is probably due to small, transient defects or fluctuations in the packing of the hydrophobic blocks at various locations on the membrane.

**Fig. 3:**
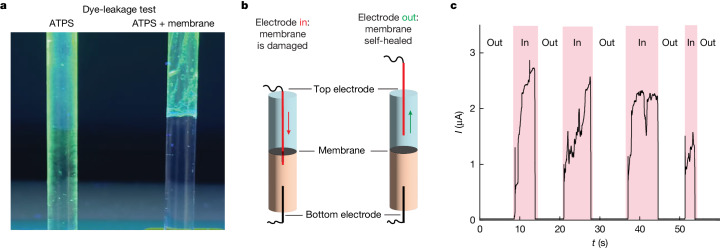
Self-healing properties of ATPS-supported BCP membranes. **a**, Dye-leakage test showing that a BCP membrane at an ATPS interface can block the diffusion of the negatively charged dye, calcein, that was added to the PEO phase. The photograph shows glass cylinders with an inner diameter of 6 mm under ultraviolet light 2 h after sample preparation at room temperature. **b**,**c**, Schematic representation (**b**) of the self-healing test plotted in **c**. We measured the current across a membrane via two Ag/AgCl electrodes at a constant applied potential of 100 mV. Initially the two electrodes were distant to the membrane (Out) and the transmembrane current was negligible, indicating that the membrane had no defects. Subsequently, we lowered the top electrode (with a cross-sectional diameter of 100 µm, approximately 3,000-fold the membrane thickness) using a precision linear positioner until it perforated the membrane (In), leading to a sudden current jump. When we retracted the electrode (Out) the current returned to zero, demonstrating that the damage inflicted by the electrode had been repaired. Four successive trials demonstrated that the membrane can heal following repeated damage. Both the thickness (capacitance) and resistance of the membrane, as determined via electric measurement, remained unaltered following each self-healing cycle. The self-healing experiment was repeated on three different membranes. All membranes were formed using BCP1 and BCP2. Source Data

Supplementary Discussions 3–5 provide an extensive experimental and theoretical characterization of ATPS-supported membranes. For instance, fluorescence resonance energy transfer experiments confirm that the membranes are indeed asymmetric, with the two BCPs partitioning in different proportions to the two sides of the bilayer as suggested for the proposed membrane formation mechanism (Fig. 1). Coarse-grained molecular dynamics (CGMD) simulations (Fig. 2b,c) and self-consistent field (SCF) computations (Fig. 2c) support this experimental result. The mechanical properties of these large-area, ATPS-stabilized membranes are comparable to those of the cell membrane^25^, with a penetration force as measured by atomic force microscopy (AFM) of approximately 4 nN (Fig. 2d). Moreover, ATPS-stabilized membranes can be made even if osmotic pressure differences of up to 4 kPa are present between the PEO and DEX phases, above which detectable defects start to form (Supplementary Fig. 17). The ATPS-supported membranes provide an excellent barrier function regarding the passage of charged organic dyes (Fig. 3a) and possess the ability to self-heal from repeated mechanical damages over a length scale of 100 µm, which exceeds their thickness by approximately 3,000-fold, as shown in Fig. 3b,c. Simulations by CGMD confirm the self-healing capability of the membranes (Supplementary Video 1) and provide an estimate of the in-plane diffusion coefficient of the BCPs, *D* ≈ 8 μm^2^ s^−1^, which is consistent with the lateral diffusivity of poly(dimethyl siloxane) (PDMS)-based BCP bilayers^26^ and confirms that the ATPS-supported membranes are fluid.

## Ion-selective, ATPS-supported membranes

**Fig. 4:**
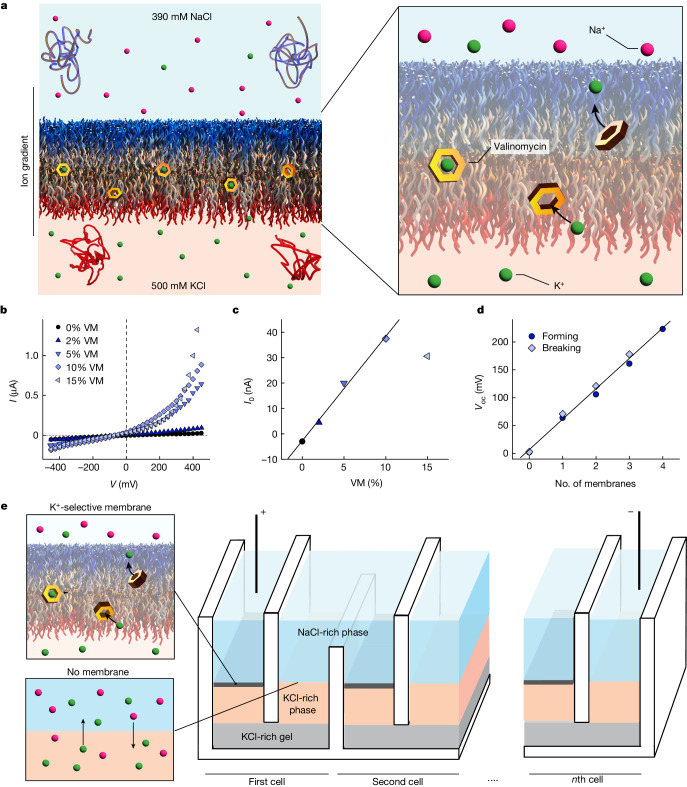
Electrical power generation by ATPS-supported, ion-selective BCP membranes. **a**, Schematic of valinomycin (VM)-doped membranes showing the selective K^+^ transport mechanism; VM is represented by yellow hexagons. **b**, Current–voltage (*I–V*) curves for membranes prepared with varying VM:BCP molar ratios. **c**, Currents (*I*_0_) extracted from the *I–V* curves in **b** for potentials *V* = 0 mV. Above 10 mol% VM, incorporation of more VM yielded no increase in *I*_0_ increase reduced the temporal stability of the membrane, suggesting that the ionophore aggregates in the membrane above 10% molar ratio or starts to induce packing defects of the BCPs in the membrane. The experiment was repeated another time (data not shown) **d**, Open-circuit voltage *V*_oc_ of the device inspired by Atlantic torpedo^28^ as a function of the number of membranes doped with 5 mol% VM. Dark blue markers and linear fits represent increases in potential difference following membrane formation. Light blue markers represent potential drops following intentional membrane disruption, which was achieved by stirring the interface of the ATPS with a syringe needle. The experiment was repeated four other times (data not shown) **e**, Schematic of an electric-ray-activated power unit with stacks of ion-selective membranes developed in this work. All membranes were formed using BCP1 and BCP2. Source Data

Biological membranes are more than barriers, because they control the transport of specific compounds with unique selectivity and efficiency. We imparted the same functionality to ATPS-supported, large-area membranes by incorporating the selective ion transporter valinomycin (Fig. 4a), which is a naturally occurring cyclic hydrophobic peptide bearing a K^+^-selective binding pocket. Its hydrophobicity promotes accumulation within the hydrophobic core of the membrane, where it can freely diffuse and shuttle K^+^ ions to equilibrate transmembrane concentration gradients^27^.

**Extended Data Fig. 1:**
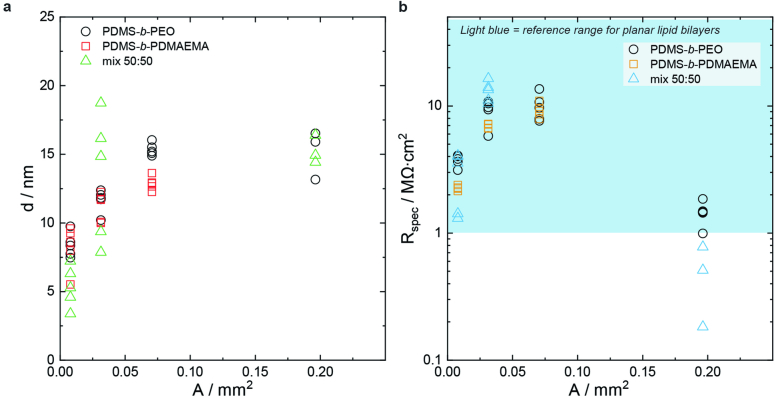
Small-area BCP membranes obtained with the planar bilayer folding method. a) Bilayer thickness (*d*) versus area (*A*) and b) Specific resistance (*R*_sp_) versus area (*A*) for membranes formed using either BCP1, BCP2 or a 50/50 wt% mix of both. Membranes were formed using Teflon films with holes of 100, 200, 300 and 500 μm diameters as support; The light blue area in b) indicates the reference range for typical lipid membranes^38,46^ formed in comparable setups.

We first explored and confirmed the incorporation of valinomycin on small-scale BCP membranes comprising BCP1 and BCP2 obtained via the conventional folding method (Extended Data Fig. 1). Subsequently we incorporated valinomycin into ATPS-supported membranes by adding the peptide to the solution of BCPs in toluene. We modified the ATPS to introduce an ion concentration gradient across the membrane (Fig. 4a), using KCl (500 mM) as an electrolyte in the DEX phase and NaCl (390 mM, to achieve osmotic matching of the phases) in the PEO phase. The gradient thus produced resembles the one across the plasma membrane of living cells (albeit with higher ion concentrations) and is expected to yield a transmembrane potential if selective ion transport occurs (Supplementary Discussion 7).

We tested various molar ratios of valinomycin to BCPs and found that the incorporation of valinomycin causes a progressive reduction in membrane electrical resistance and the appearance of potential and current offsets, as expected^27^ (Fig. 4b–d). These measurements demonstrate that the ATPS-supported BCP membranes presented here are fluid because ionophores can freely diffuse in their hydrophobic core to shuttle ions, and that they can be transformed into macroscopic ion-selective membranes. Ion selectivity is very difficult to achieve in bulk materials but allows conversion of the energy from isotonic ion gradients (for example, 1 M KCl/1 M NaCl). Quasi-isotonic NaCl/KCl gradients are ubiquitous in biology, with sodium and potassium pumps maintaining a cell environment rich in KCl and expelling NaCl to the extracellular space.

## Towards biomimetic power generation

**Extended Data Fig. 2:**
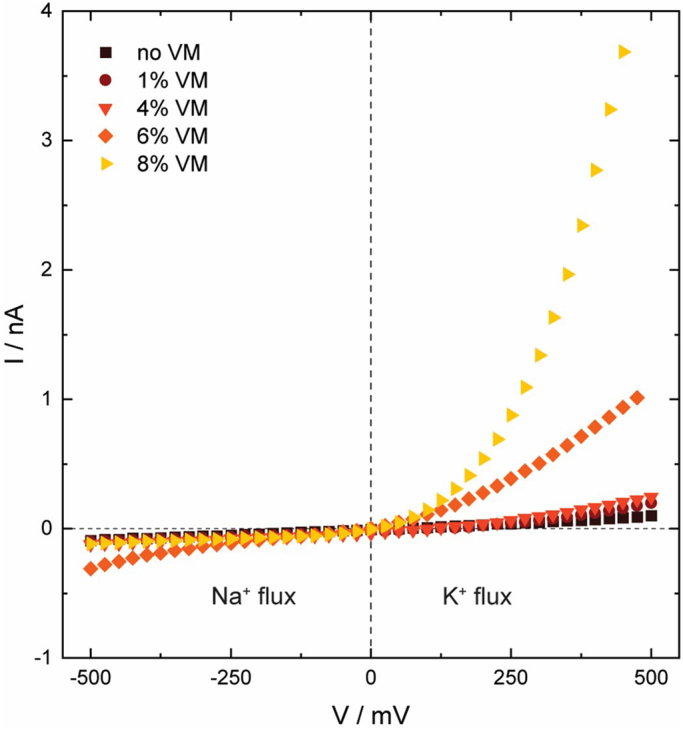
Transmembrane current as a function of applied voltage with varying concentrations of valinomycin. (mol% with respect to the block copolymers). Membranes made of BCP1 and BCP2 were formed with the planar bilayer folding method across a hole with 100 μm diameter. Salt concentrations in the two chambers of the setup were 1 M NaCl and 1 M KCl.

The Atlantic torpedo (*Tetronarce nobiliana*), a species of electric ray, uses ion selectivity to convert isotonic ion gradients into electric discharges of up to 1 kW power^28^. This fish evolved plate-like cells (electrocytes) that behave like a small battery, thanks to a special enervated membrane^28^. The membrane at one side of the cell can generate a potential by opening selective ion channels on demand. The opposite side does not contribute to the potential but has a low resistance to ensure a high ionic current flow. Stacking electrocytes in series and parallel allows the torpedo ray to produce biology’s strongest output of electrical current and power (see schematic in Supplementary Fig. 2). Inspired by the torpedo ray^29,30^, we designed a small electric power generator that takes advantage of the unique features of an ATPS to electrically stack multiple ion-selective membranes in series. As shown in Fig. 4e, the fundamental unit of the device consists of two ATPSs, which are ionically connected via the DEX (+500 mM KCl) phases through a KCl-rich hydrogel bridge (Supplementary Methods). The hydrogel ensures high conductivity and prevents hydrostatic mismatch of communicating vessels. We formed a valinomycin-doped BCP membrane (with a diameter of 6 mm) at one of the two interfaces with the solvent-displacement method but we did not form a membrane at the other interface, to facilitate the passage of ions with minimal resistance (Fig. 4e). These two interfaces imitate the two sides of torpedo rays’ electrocytes. Figure 4d shows that membrane potentials become additive when several fundamental units are connected via the PEO phases and that the open-circuit voltage (*V*_oc_) of the device scales linearly with the number of membranes. Each additional unit contributes to the overall potential difference with approximately 60 mV, similar to the potential generated by the torpedo’s electrocytes, although larger potentials were obtained in some attempts (Extended Data Fig. 2). Intentionally breaking the membranes mechanically caused a stepwise drop in potential difference, with reductions of approximately 60 mV. This observation confirms that the potential difference of the device originates from additive transmembrane potentials. By solving the Goldman–Hodgkin–Katz equation^31,32^, which relates *V*_oc_ to ion concentrations and membrane permselectivity for the various ionic species, we determined that the valinomycin-doped membrane is approximately eight times more permselective for K^+^ than for Na^+^ (Supplementary Discussion 7), in agreement with previous reports^33^.

The device was not optimized for power output (power density per membrane, PD ≅ 0.04 mW m^−2^) because K^+^ transport mediated by valinomycin is not efficient, as verified by the low current (*I*_0_) values in Fig. 4c. In future, the use of natural or synthetic ion channels, which can be 100- to 1,000-fold more efficient in transporting ions than valinomycin^34–36^, may enhance device performance up to that of the electric ray (PD ≅ 28 W m^−2^)^28^. Because BCP membranes have been used for the reconstitution of functional transmembrane proteins^37^, the final step in realizing a continuously active artificial electric organ can now be envisaged, namely the fabrication of stable, large-area, ion-selective membranes that use chemical fuels such as ATP to produce electric power. The planar nature of these membranes makes it possible to stack them in series to reach the desired potential differences. These macroscopic membranes may also find application in electrophysiology and other biophysical characterization methods of membrane proteins, such as X-ray or neutron reflectivity. Finally, these membranes may enable high-value separations, blue power generation and the preparation of ion-selective membranes for fuel cells.

## Methods

### Electric characterization of self-assembled membranes

The electric behaviour of self-assembled bilayers is described by an equivalent circuit that consists of a resistor and capacitor in parallel^23^. The resistive part describes the ability of the bilayer to hinder the passage of ions and, therefore, quantifies its barrier function. The capacitive element is related to the hydrophobic core of the bilayer, which acts as a dielectric material between two conductive regions, similar to a plate capacitor. The behaviour of such a system is described by

1 $$I=A\left[\frac{V}{{R}_{{\rm{sp}}}}+\left(\frac{\varepsilon {\varepsilon }_{0}}{d}-\frac{{C}_{{\rm{S}}}}{A}\right)\frac{{\rm{d}}V}{{\rm{d}}t}\right],$$

where *I* (A) is the current, *V* (V) the applied potential, *ε*_0_ the vacuum permittivity, *t* (s) the time, *C*_S_ (F) the capacitance of the set-up excluding the membrane, *A* (m^2^) the area of the bilayer, *d* (m) the thickness of the hydrophobic core, *R*_sp_ (Ω) the specific resistance of the membrane and *ε* the relative dielectric constant of the hydrophobic core. According to equation (1), *d* and *R*_sp_ can be determined from electric measurements that apply time-varying potentials. We used a value of 2.7 for *ε* (refs. ^23,40^).

An e2HC amplifier (Elements Srl) connected to two Ag/AgCl electrodes was used to measure currents in response to applied voltage protocols. For the characterization of ATPS-stabilized membranes formed in the presence of 500 mM KCl in the DEX phase and 390 mM NaCl in the PEO phase, we accessed the compartments via salt bridges (1 M KCl in acrylamide gel) that contained the Ag/AgCl electrodes. Without these salt bridges, an offset potential of approximately 4 mV would have been present resulting from the slightly different concentrations of Cl^−^ ions in the DEX and PEO phases. The system was placed on an anti-vibration table inside a Faraday cage to prevent electrostatic and vibrational noise from affecting the measurements. The e2HC amplifier makes it possible to apply square-wave voltage protocols to determine *R*_sp_, and triangular-wave voltage protocols to determine *d* independently. When the bilayer folding set-up was used, the capacitance of the set-up *C*_S_ was determined by putting a Teflon partition (without a hole) between the two chambers filled with electrolyte solution. The measured capacitance was *C*_S_ = 25 pF, and this value was deducted for all measurements of the membranes. By contrast, the capacitance of the ATPS set-up was ignored because it is much smaller than that of the formed bilayer (*εε*_0_/*d* ≫ *C*_S_/*A*). All recordings were analysed with a custom-made Python code to determine *d* and *R*_sp_.

### Composition of BCPs

The solvent-displacement method uses two different BCP types with the same hydrophobic but different hydrophilic blocks that extend into the DEX and PEO phases, respectively. Naturally, PEO and DEX are ideal choices for the hydrophilic blocks because the PEO block will favourably interact with the aqueous PEO phase and the DEX block will favourably interact with the aqueous DEX phase. However, although PEO-based BCPs are relatively straightforward to prepare^41^, DEX is difficult to functionalize selectively, typically has a broad molecular weight distribution and is almost exclusively soluble in water. Encouraged by previous reports on the use of PDMAEMA-based BCPs to stabilize PEO/DEX water-in-water emulsions^42,43^, we selected PDMAEMA as the second hydrophilic block. We tested poly(hexyl methacrylate) and PDMS as a common hydrophobic block for the two BCP types and we found that they both enabled the formation of large-area, ATPS-supported membranes (Supplementary Discussion 2). We chose PDMS for most of the experiments, however, because it is inert, non-toxic and shows a low glass transition temperature^44^. We also expected these characteristics to be favourable for the formation of fluid, self-healing membranes.

**Extended Data Fig. 3:**
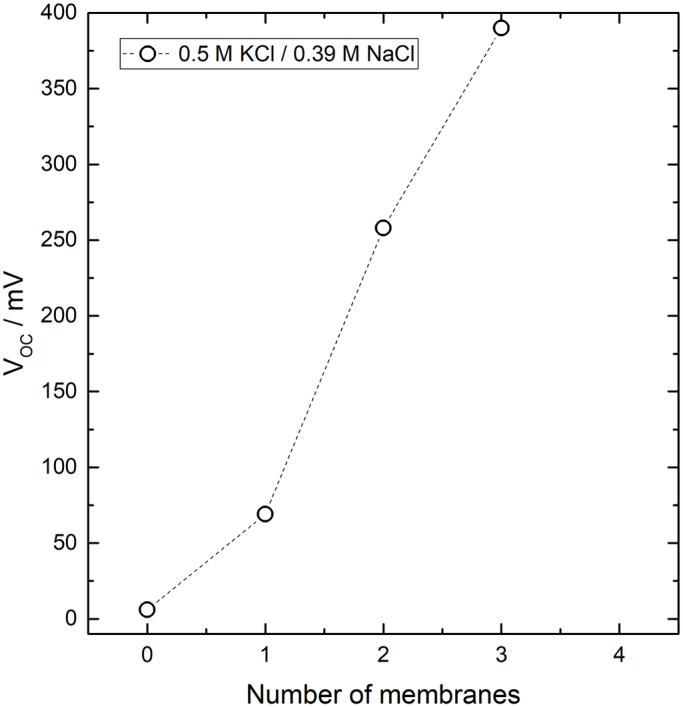
Exceptionally high open-circuit voltages. For three membranes in series, containing 5 mol% VM. The fourth membrane in the setup broke during preparation. The average open circuit voltage *V*_OC_ was approximately 130 mV per cell.

We synthesized and characterized BCPs of composition PEO_2kDa_-*b*-PDMS_5kDa_ (BCP1) and PDMAEMA_2kDa_-*b*-PDMS_5kDa_ (BCP2), as described in Supplementary Methods, and confirmed their ability to form planar bilayers using the well-established vertically folded planar bilayer method^14,23^ (Supplementary Methods, Extended Data Fig. 3 and Supplementary Discussion 2). However, producing membranes of area larger than 0.2 mm^2^ proved challenging, reflecting the fact that this conventional method for the formation of planar bilayers is not suitable for the preparation of macroscopic membranes.

### Solvent-displacement method for the formation of large-area membranes

The BCP membranes, supported by an ATPS, were formed using osmotically matched solutions of PEO, DEX and salt. For a system containing only one type of salt (NaCl), 197 mg ml^−1^ DEX and 120 mg ml^−1^ PEO were dissolved in a 1 M NaCl solution. The phase-separated mixture was left to equilibrate overnight, after which the two phases were isolated by means of a separation funnel. For an ATPS with two types of salt (NaCl in the PEO phase and KCl in the DEX phase), the osmolality of 197 mg ml^−1^ DEX in 0.5 M KCL was measured (885 mOsm kg^−1^) and the osmotically matching NaCl concentration (0.39 M) for the 120 mg ml^−1^ PEO solution was determined from a calibration curve (Supplementary Methods and Supplementary Fig. 3).

**Extended Data Fig. 4:**
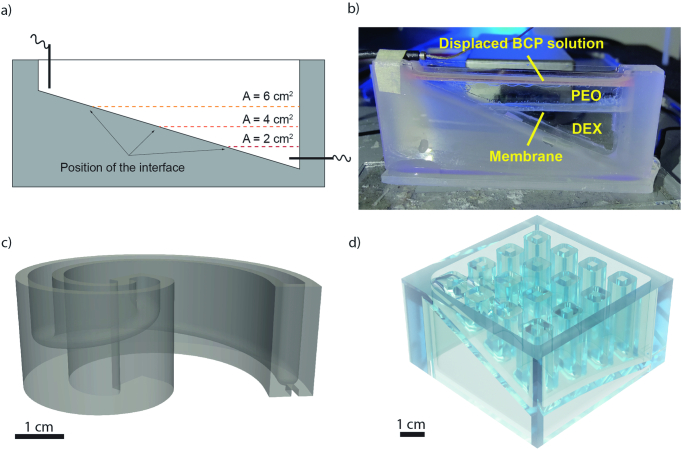
Containers used for the preparation of macroscopic scale ATPS-supported membranes. a) Schematic illustrating how elongated cells with slanted floor make it possible to control membrane area by varying the position of the ATPS interface. b) ATPS-supported membrane formed by BCP1 and BCP2 using an elongated cell with slanted floor. The displaced toluene solution is floating on top of the PEO phase while a membrane is formed at the PEO/DEX interface. c) 3D rendering of the spiral set-up with slanted floor that allowed to form BCP membranes of up to 8 cm^2^ surface area. d) 3D rendering of the set-up with internal supporting pillars and slanted floor used to form membranes with 10 cm^2^ surface-area.

To construct the membrane, a containing vessel, such as a glass Pasteur pipette of 4.5 mm internal diameter, was cut to a few centimetres in length and closed at the bottom with a rubber plug in which an Ag/AgCl wire electrode was embedded. Then, 0.5 ml of DEX/salt solution was pipetted into the glass cylinder followed by the addition of 50 μl of BCP solution in toluene. Because the mass concentration of each BCP was 25 mg ml^−1^, the total BCP concentration was 50 mg ml^−1^. After 5 min, 0.5 ml of PEO/salt solution was injected by syringe and needle, just below the surface of the toluene solution near the side of the glass wall. This gentle injection expelled the toluene to the top, producing a polymer membrane at the newly formed PEO–DEX interface. Excess toluene was removed from the top by means of a syringe, and a second Ag/AgCl wire electrode was inserted in the top phase. The electrodes were then connected to the amplifier to characterize the membrane. Other glass containers of different diameter (*D*_int_) that were used to assess membranes with smaller and larger areas included NMR tubes (*D*_int_ = 3.4 mm) and various types of culture tube (*D*_int_ = 8.0, 10.4 and 13.4 mm). To further extend the membrane area, we three-dimensionally printed resin containers with a straight channel and a spiralling channel (Extended Data Fig. 4). The internal width of these channels was 5 mm and the bottom of the containers was slanted, so that the membrane area could be tuned by depositing less or more of the DEX solution in the bottom. Wire electrodes were inserted from the bottom at the deepest end and the counter electrodes were submerged in the PEO solution from the top following membrane assembly. The slanted bottom of the containers enabled gentle expulsion of excess toluene solution when injecting the PEO top solution from the higher part of the slanted bottom. To form membranes with surface area of 10 cm^2^, we used a container with internal pillars to support the membrane (Extended Data Fig. 4). We observed good adhesion of the edge of the membranes in both glass containers and in those made of resin. We suggest that the key to ensuring such adhesion, which is crucial in avoiding lateral leakage, is to use materials for the construction of cells that are chemically compatible with the hydrophobic blocks of the copolymers.

### Incorporation of valinomycin in BCP membranes

To incorporate valinomycin into BCP membranes, a stock solution was prepared containing 25 mg ml^−1^ of each BCP and 1.8 mg ml^−1^ valinomycin (1.62 mM, 20 mol% with respect to the total amount of BCP) in toluene. To obtain the desired final concentration of valinomycin, this stock solution was mixed in the corresponding ratio with a BCP solution without any valinomycin. To check the incorporation and function of valinomycin in the membranes, the planar bilayer set-up (with 110 μm aperture) was again used, this time with DEX/KCl and PEO/NaCl solutions in the respective chambers rather than equimolar KCl solutions (Extended Data Fig. 2). Large-area membranes containing valinomycin were then formed by the solvent-displacement method using valinomycin-containing BCP solutions in toluene. *I*–*V* curves were recorded by stepwise increase or decrease of the voltage between −500 and +500 mV with steps of 25 mV. Each step was recorded for a duration of 5 s. The corresponding current value was determined by discarding the first 500 ms of the step, because of transient capacitive currents induced by the stepwise potential change.

## Online content

Any methods, additional references, Nature Portfolio reporting summaries, source data, extended data, supplementary information, acknowledgements, peer review information; details of author contributions and competing interests; and statements of data and code availability are available at 10.1038/s41586-024-07481-2.

### Supplementary information

Supplementary Information This file contains Supplementary methods, Discussions 1–7, Tables 1–6 and Figs. 1–29.

Supplementary Video 1 A molecular dynamics simulation run that shows the damage induced by lateral stresses and subsequent self-healing mechanism of a self-assembled BCP membrane stabilized by the interface of an ATPS.

### Source data

Source Data Fig. 2

Source Data Fig. 3

Source Data Fig. 4

## Data Availability

The data supporting the findings in this work are freely available through Zenodo at 10.5281/zenodo.7818212 (ref. ^45^). Source data are provided with this paper.

## Code availability

The Python code used for the data analysis is freely available through Zenodo at 10.5281/zenodo.7818212 (ref. ^45^).
