# Supplementary material for: Large-area, self-healing block copolymer membranes for energy conversion
Source: Nature. 2024 Jun 5;630(8018):866–71. doi: 10.1038/s41586-024-07481-2 (PMC11208134; doi:10.1038/s41586-024-07481-2)
Supplement: Supplementary file 1 — This file contains Supplementary methods, Discussions 1–7, Tables 1–6 and Figs. 1–29. [file 41586_2024_7481_MOESM1_ESM.pdf]

nature portfolio

https://doi.org/10.1038/s41586-024-07481-2

Supplementary information

Large-area, self-healing block copolymer membranes for energy conversion

In the format provided by the authors and unedited

Supplementary information to:

Large-area, self-healing block copolymer membranes for energy conversion

Christian C. M. Sproncken, Peng Liu, Justin Monney, William S. Fall, Carolina Pierucci, Philip B. V. Scholten, Brian Van Bueren, Marcos Penedo, Georg Ernest Fantner, Henricus H. Wensink, Ullrich Steiner, Christoph Weder, Nico Bruns, Michael Mayer, Alessandro Ianiro**

*corresponding authors: alessandro.ianiro@unifr.ch; michael.mayer@unifr.ch

Contents

A. Supplementary Methods 3
1. Materials 3
2. Instrumentation 3
3. Experimental methods 4
Synthesis of PDMS-b-PEO and PDMS-b-PEO-Cy3 4
Synthesis of PDMS-b-PDMAEMA 5
Synthesis of PHMA-b-PEO 6
Synthesis of PHMA-b-PDMAEMA 6
Preparation of Ag/AgCl electrodes 7
Bilayer folding method 7
Osmotically matching DEX and PEO in salt solutions 8
Preparation of the dry AFM samples 8
Electric device with cells in series 8
B. Supplementary Discussion 10
1. Thermodynamics of asymmetric membrane formation at the ATPS interface 10
2. Control experiments 11
Membranes prepared with the folding method 11
Control experiments with the ATPS 11
Membranes with PHMA as hydrophobic blocks 11
Control experiment with a peptide that is not an ionophore 11
3. Characterization of the membranes formed at the ATPS interface 12
Evolution over time and thickness 12
Reproducibility 12
Surface area and stability 12
Permeability 13

Mechanical properties 13
Membrane asymmetry.. 14
4. Coarse-grained (CG) molecular dynamics simulations 15
Methodology 16
Concentration Profiles.. 17
Mean-Squared Displacement and Diffusion 17
Self-Healing 18
5. Self-consistent field studies of ATPS-supported membranes 18
Membrane asymmetry.. 19
Membrane thickness.. 20
6. Viscosity requirements of the solvent displacement method 21
7. Membrane permselectivity ratio and device power output.. 21
Voltage output over time.. 22
C. Supplementary Tables and Figures.. 23
D. Supplementary References 58

A. Supplementary Methods

1. Materials

Sodium chloride (>99.5 %), potassium chloride (>99.5 %), silver wire (>99.99 %, D = 0.25 mm) hexadecane (>99 %), methanol, dextran from *Leuconostoc* spp. (M_r = 450-650 kDa), poly(ethylene oxide) (M_n = 35 kDa), poly(ethylene glycol) methyl ether (PEO, 2000 Da), valinomycin (>99 %), alamethicin, 2,2′-azobis(isobutyronitrile) (AIBN, >98 %), 4-cyano-4-(phenyl carbonothioylthio)pentanoic acid (chain transfer agent, 'CTA'), 2-(dimethylamino)ethyl methacrylate (DMAEMA), hexyl methacrylate (HMA), succinic anhydride, anhydrous pyridine (>98%), 4-dimethylaminopyridine (DMAP), 1-ethyl-3-(3-dimethylaminopropyl)carbodiimide (EDCI), calcein, 2-hydroxy-4′-(2-hydroxyethoxy)-2-methylpropio-phenone, and monohydroxy terminated poly(dimethyl siloxane) (PDMS, 4650 Da) were purchased from Sigma-Aldrich (Merck KGaA, Germany). Cy5 and COOH-PEO(2 kDa)-Cy3 were from BroadPharm. HMA and DMAEMA were passed through a short basic alumina plug before use. *n*-Hexane, isopropyl alcohol (IPA, 99%), chloroform (99%), dichloromethane (DCM, 99%) and methyl *tert*-butyl ether were purchased from Reactolab SA (Switzerland). Toluene (AR-certified) was obtained from Fisher Scientific AG (Switzerland). Ultrapure water (18 MΩ·cm) was obtained from a Purelab Flex purification system (Veolia ELGA, Germany). Teflon partitions with defined hole size were purchased from Eastern Scientific LLC (Rockville, MD, USA). The exact hole sizes in the partitions as measured using an optical microscope with 2 μm precision were as follows: 108, 110, 112 (referred to as 110 μm); 194, 208 (referred to as 200 μm); 286, 290 (referred to as 290 μm); 480 and 488 μm (referred to as 490 μm).

2. Instrumentation

Size exclusion chromatography (SEC). Relative molecular weights and molecular weight distributions of polymers were measured by SEC on a 1200 series HPLC system (Agilent Technologies, Inc., USA), equipped with an Agilent PLgel mixed guard column (particle size = 5 μm) and two Agilent PLgel mixed-D columns (ID = 7.5 mm, L = 300 mm, particle size = 5 μm). Signals were recorded by an UV detector (Agilent 1200 series, 346 nm), an Optilab REX interferometric refractometer, and a miniDawn TREOS light scattering detector (Wyatt Technology Corp., USA). Samples were eluted using THF at 30 °C and a flow rate of 1.0 mL min^{-1}. The molecular weight was determined based on the calibration of poly(methyl methacrylate) standards. The sample solutions were filtered through a 13 mm syringe filter (Nylon 66, nonsterile, pore size 0.22 μm, Swiss Labs) before injection.

^{1}H-NMR spectroscopy. ^{1}H-NMR spectra were recorded on an Avance III 400 MHz NMR spectrometer (Bruker, USA). $CDCl_3$ was used as deuterated solvent for all the polymers. Data were evaluated with the MestReNova software suite and all chemical shifts (δ) are reported in parts per million (ppm).

Electric measurements. An e2HC amplifier (Elements S.R.L. Italy) was used to record transmembrane currents, controlled with the designated EDR4 software and placed in a Faraday cage on a spring-operated antivibration table. A current range of 4 μA and a sampling rate of 62.5 kHz were used. Generally, triangular voltage waves with an amplitude of 50 mV and a time period of 100 ms were applied for determination of capacitance. A model 2450 source meter (Keithley, USA) was used as power supply during Ag/AgCl electrode fabrication, as well as to record voltages (open-circuit and under load) of ion-selective membrane-based electric cells.

Dry atomic force microscopy. Dry AFM was conducted on an NX10 atomic force microscope (Park Systems, Republic of Korea), operated with SmartScan software in tapping mode, scanning 1024 x 1024 pixels (26.5 x 26.5 μm) at 0.25 Hz at 16 nm setpoint. The cantilevers were OMCL-AC160TS (Olympus Corporation, Japan) with 7 nm tip radius, force constant of 26 Nm^{-1} and 300 kHz resonance frequency. During the measurements, a 32 nm amplitude, 3.03 % drive and 299.12 kHz frequency were used. The

recorded images were further analyzed with XEI (Park Systems, version 5.2.4). Average membrane thickness was calculated from line profiles in 6 different locations on the sample.

Liquid-phase atomic force microscopy. AFM imaging in liquid was performed on a custom-built setup composed by a SPM controller and its related software (LabView)[3] and a custom-built high voltage amplifier combined with a E-scanner attached to a commercial Multimode AFM base. Information about the different AFM components can be found on the Open Hardware project web page (https://www.epfl.ch/labs/lbni/openhardware/). Tapping mode AFM imaging was performed using ScanAsyst-Fluid+ cantilevers from Bruker, with a nominal spring constant of 0.7 N/m. Setpoint was set to around 40% of the cantilever's free amplitude (20 nm) due to the viscous behavior of the membrane, at a frequency of 67 kHz. Scan rate was set to 300 mHz, and the scan area was 8 µm x 8 µm (256 x 256 pixels^2). The force versus distance curve was acquired vertically moving the cantilever at 200 nm s^{-1}, using trigger distance control. AFM imaging was performed in water environment.

AFM images were processed in Gwyddion, where plane leveling, median line flatten, and scar correction filters were used during the image processing.

Osmotic pressure measurements. A model 3320 micro-osmometer (Advanced Instruments, USA) was used to measure the osmolality of aqueous polymer/salt solutions. The sample volume was 20 µL.

Fluorescence resonance energy transfer spectroscopy

Fluorescence resonance energy transfer measurements were performed using a Picoquant MicorTime 200 equipped with two Solea white lasers. The membranes were prepared in special sample holders with transparent bottom obtained by gluing a glass tube on a microscopy slide. Two Ag/AgCl electrodes were placed at the bottom and the top of the glass tube. These electrodes, connected to an e2HC amplifier (Elements S.R.L. Italy) were used to measure transmembrane currents. To reduce the noise, the cell was covered with a grounded aluminum-coated box acting as a Faraday cage (**Supplementary Figure 4**).

3. Experimental methods

Synthesis of PDMS-b-PEO and PDMS-b-PEO-Cy3

For poly(dimethylsiloxane)$_{63}$-*block*-poly(ethylene oxide)$_{45}$, first, 4 g of PEO (M_n = 2000 Da, 2 mmol) were dissolved in 50 mL anhydrous pyridine. Subsequently, 2 g of succinic anhydride (20 mmol) was added to this solution which was kept in an ice bath. The mixture was then stirred for 24 h at room temperature. After that, the polymer was precipitated in methyl *tert*-butyl ether which was kept in an ice bath. The polymer suspension was then centrifuged at 5000 *g* for 10 min at 0 °C, followed by redissolution in DCM and reprecipitation in methyl *tert*-butyl ether which was kept in an ice bath. This process was repeated three times. After drying under high vacuum, a pure functionalized polymer (PEO-COOH, **Supplementary Figure 5** shows ^{1}H-NMR spectrum) was obtained (**Supplementary Figure 5 B;** PEO-OH, $M_{n(GPC)}$ = 2200 Da, $Đ$ = 1.05; PEO-COOH, $M_{n(GPC)}$ = 1700 Da, $Đ$ = 1.1). The slight shift in retention time of PEO-COOH was due to the interaction of carboxylic acid and SEC columns. To synthesize the block copolymer, 200 mg of PEO-COOH (0.1 mmol), 470 mg of PDMS-OH (M_n = 4650, 0.1 mmol) and 6.1 mg of DMAP (0.05 mmol) were dissolved in 2 mL dry DCM under Ar. Then, the solution was placed in an ice bath. EDCI (38 mg, 0.2 mol) was added into the solution in several steps under Ar. The resulting mixture was left to warm up to room temperature and was stirred for 24 h. The polymer was precipitated in a 60 vol% methanol/water mixture which was kept in an ice bath. The polymer suspension was then centrifuged at 5000 *g* for 10 min at 0 °C. The polymer was redissolved in DCM and this process was repeated three times. After drying under high vacuum, the pure copolymer

was obtained (SEC trace shown in **Supplementary Figure 5 C**). From SEC: PDMS-OH, M_n = 5700 Da, $Đ$ = 1.09, PDMS-*b*-PEO, M_n = 7600 Da, $Đ$ = 1.07 (**Supplementary Figure 5 D**).

Scheme S1. Synthesis of PEO-COOH

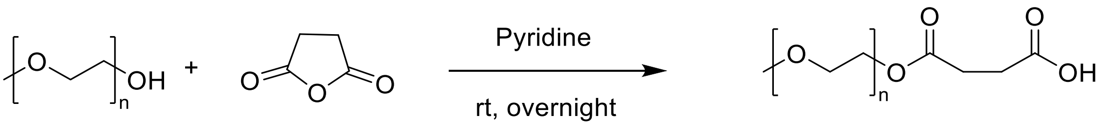

Scheme S2. Synthesis of PDMS-*b*-PEO

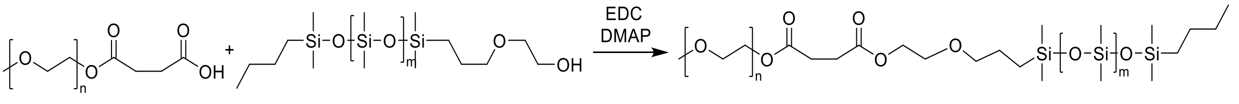

For the synthesis and purification of PDMS-b-PEO-Cy3, we followed the same procedure using COOH-PEO(2 kDa)-Cy3 for the coupling reaction. The reaction did not proceed to completion, likely because of a low purity of the commercial COOH-PEO(2 kDa)-Cy3, but enough product was obtained to perform the desired characterization. The GPC trace of the reaction crude and the purified product are shown in Figure 5E.

Synthesis of PDMS-b-PDMAEMA

To synthesize poly(dimethylsiloxane)$_{63}$-*block*-poly(dimethylaminoethyl methacrylate)$_{12}$, 470 mg of PDMS-OH (M_n = 4650 Da, 0.1 mmol), 4-cyano-4-(phenyl carbonothioylthio)pentanoic acid (chain-transfer agent, "CTA", 140 mg, 0.5 mmol) and 6.1 mg of DMAP (0.05 mmol) were dissolved in 2 mL dry DCM under Ar, after which the solution was placed in an ice bath. EDCI (96 mg, 0.5 mmol) was added into the solution in several steps under Ar. The resulting mixture was let to warm up to room temperature and was stirred for 24 h. After that, the polymer was precipitated in methanol which was kept in an ice bath. The polymer suspension was then centrifuged at 5000 *g* for 10 min at 0 °C. The polymer was redissolved in DCM and this process was repeated three times. Drying under high vacuum yielded a pure functionalized polymer. For the block copolymer, the PDMS-CTA (430 mg, 0.09 mmol) was dissolved in 2 mL 1,4-dioxane, along with the initiator, AIBN (3 mg, 0.018 mmol). The monomer 2-(dimethylamino)ethyl methacrylate (DMAEMA) was purified to remove the quinone-based inhibitors by passing it through a basic aluminum oxide plug. Then, purified DMAEMA (184 mg, 1.17 mmol) was added to the AIBN and CTA solution. The mixture was bubbled for 1 h with Ar. The molar ratio I:CTA:M was 1:5:65. The reaction was initiated by heating the solution to 65 °C under Ar. The reaction was terminated after 16 h by exposing the solution to atmospheric oxygen. The polymer was precipitated in a 60 vol% methanol/water mixture which was kept in an ice bath. The polymer suspension was then centrifuged at 5000 *g* for 10 min at 0 °C. The polymer was dissolved again in DCM. This was repeated three times, followed by drying under high vacuum to yield a pure copolymer (^{1}H-NMR spectrum, **Supplementary Figure 6 A**; SEC, **Supplementary Figure 6 B**, M_n = 8100 Da, $Đ$ = 1.14).

Scheme S3. Synthesis of PDMS-CTA

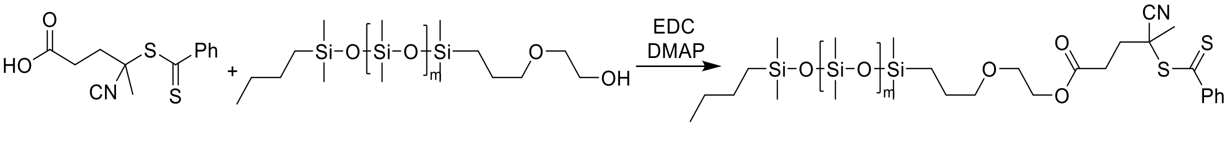

Scheme S4. Synthesis of PDMS-*b*-PDMAEMA

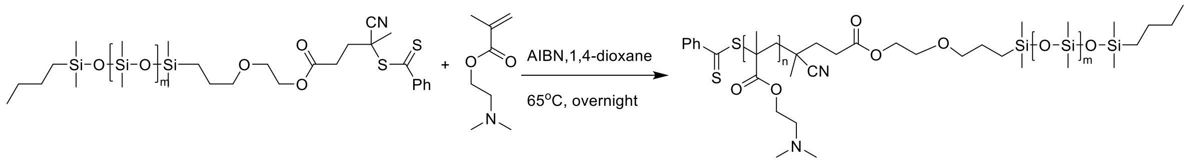

Synthesis of PHMA-b-PEO

To synthesize poly(hexyl methacrylate)$_{65}$-*block*- poly(ethylene oxide)$_{45}$, 2 g of PEO (M_n = 2000 Da, 1 mmol), CTA (1.4 g, 5 mmol) and 61 mg of DMAP (0.5 mmol) were dissolved in 10 mL dry DCM under Ar, after which the solution was placed in ice bath. EDCI (960 mg, 5 mmol) was added into the solution in several steps under Ar. The resulting mixture was let to warm up to room temperature and was stirred for 24 h. After that, the polymer was precipitated in methyl *tert*-butyl ether which was kept in an ice bath. The polymer suspension was then centrifuged at 5000 *g* for 10 min at 0 °C. The polymer was re-dissolved in DCM and re-precipitated in methyl *tert*-butyl ether which was kept in an ice bath. This precipitation was repeated three times. After drying under high vacuum, a pure functionalized polymer was obtained. The PEO-CTA (500 mg, 0.25 mmol) was dissolved in 5 mL 1,4-dioxane, along with the initiator, AIBN (8.2 mg, 0.05 mmol). Then, fresh purified HMA (1.5 g, 8.8 mmol) was added to the AIBN and CTA solution. The mixture was bubbled for 1 h with Ar. The molar ratio Initiator:CTA:Monomer was 1:5:176. The reaction was initiated by heating the solution to 65 °C under Ar. The reaction was terminated after 16 h by exposing the solution to atmospheric oxygen. The polymer was precipitated in a 60 vol% methanol/water mixture which was kept in an ice bath. The polymer suspension was then centrifuged at 5000 *g* for 10 min at 0 °C. The polymer was redissolved in DCM and reprecipitated in a 60 vol% methanol/water mixture which was kept in an ice bath. This was repeated three times. After drying under high vacuum, a pure polymer was obtained (^{1}H-NMR spectrum, **Supplementary Figure 7 A**; SEC, **Supplementary Figure 7 B**, M_n = 13000 Da, $Đ$ = 1.07).

Scheme S5. Synthesis of PEO-CTA

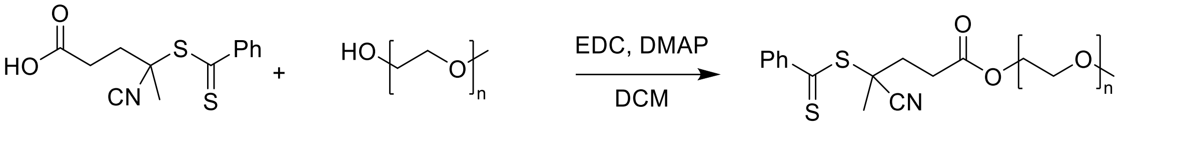

Scheme S6. Synthesis of PHMA-*b*-PEO

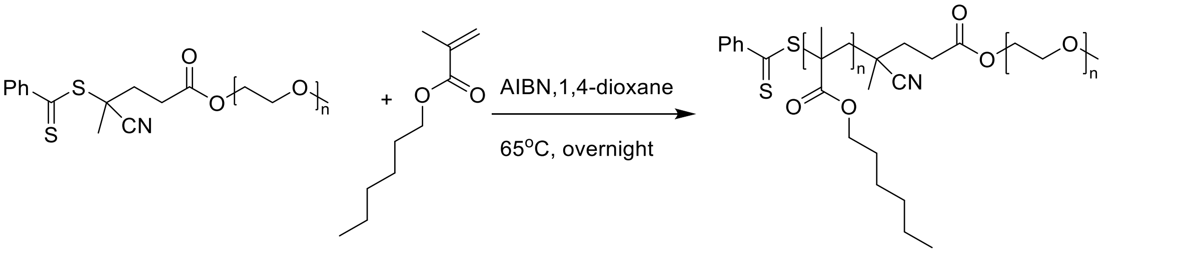

Synthesis of PHMA-b-PDMAEMA

To synthesize poly(hexyl methacrylate)$_9$-*block*-poly(dimethylaminoethyl methacrylate)$_9$, firstly CTA (164 mg, 0.56 mmol) was dissolved in 2.5 mL 1,4-dioxane, along with the initiator, AIBN (9 mg, 0.056 mmol). Then, fresh purified DMAEMA (1.2 g, 7.6 mmol) was added to the AIBN and CTA solution. The mixture was bubbled for 1 h with Ar. The molar ratio Initiator:CTA:Monomer was 1:10:130. The reaction was initiated by heating the solution to 65 °C under Ar. The reaction was terminated after 20 h by exposing the solution to atmospheric oxygen. The polymer was precipitated in methyl *tert*-butyl ether

which was kept in an ice bath. The polymer suspension was then centrifuged at 5000 *g* for 10 min at 0 °C. The polymer was re-dissolved in DCM and re-precipitate in methyl *tert*-butyl ether. This was repeated three times. After drying under high vacuum, a pure polymer was obtained ($M_{n(theory)}$ = 2000 Da, $M_{n,SEC}$ = 400 Da, $Đ$ = 1.4, $M_{n(1H\ NMR)}$ = 1500 Da; the difference in molecular weight from SEC of PDMAEMA-CTA could be caused by the interaction of amine and the column). Then, PDMAEMA-CTA (500 mg, 0.33 mmol) was dissolved in 2 mL 1,4-dioxane, along with the initiator, AIBN (10 mg, 0.066 mmol). Then, fresh purified HMA (1.02 g, 6 mmol) was added to the AIBN and CTA solution. The mixture was bubbled for 1 h with argon. The molar ratio Initiator:CTA:Monomer was 1:5:90. The reaction was initiated by heating the solution to 65 °C under argon. The reaction was terminated after 20 h by exposing the solution to atmospheric oxygen. Unreacted HMA monomer was still present in the reaction mixture after polymerization, as observed by NMR spectroscopy, resulting is a relatively short PHMA block. The polymer was precipitated in a 60 vol% methanol/water mixture which was kept in an ice bath. The polymer suspension was then centrifuged at 5000 *g* for 10 min at 0 °C. The polymer was re-dissolved in DCM and re-precipitate in a 60 vol% methanol/water mixture which was kept in an ice bath. This was repeated three times. After drying under high vacuum, a pure polymer was obtained (^{1}H-NMR spectrum, **Supplementary Figure 8 A**; SEC, **Supplementary Figure 8 B**, M_n = 3000 Da, $Đ$ = 1.2).

Scheme S7. Synthesis of PDMAEMA-CTA

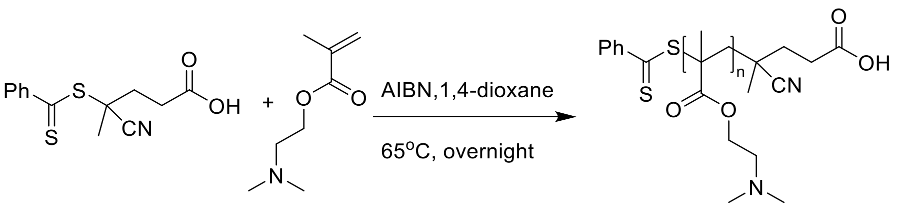

Scheme S 8. Synthesis of PHMA-*b*-PDMAEMA

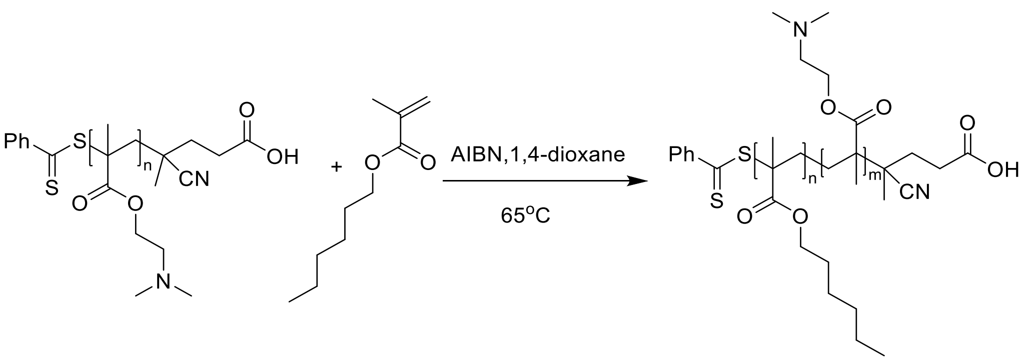

Preparation of Ag/AgCl electrodes

Pieces of silver wire, with 0.25 mm diameter, were cut to 3 cm length and scrubbed with sandpaper before suspending them in a 1 M KCl solution. A platinum wire with 0.5 mm diameter was used as a counter electrode and suspended in the same solution as the silver wire. A potential of 3.0 V was applied across the two wires while limiting the current to 10 mA. Within 30 s, the silver wire had turned dark, hinting at the formation of the AgCl and Ag2O on its surface, and the potential was removed. The Ag/AgCl electrodes were always prepared in pairs, resulting in a potential offset smaller than 1 mV between them.

Bilayer folding method

A Teflon block with two chambers, separated by a wall with a V-shaped opening, was used as a support for the thin Teflon partition used as a support for the BCP bilayers (**Supplementary Figure 9**). First, the Teflon chambers and partition were rinsed with chloroform and then the partition was fixed to the

separating wall using silicone vacuum grease. The hole in the partition was pretreated by applying 0.75 µL of 2% hexadecane solution in hexane. The two chambers were filled with equimolar salt solutions, generally 0.5 or 1.0 M KCl, until the liquid level reached below the hole in the partition. Then, 20-50 µL of BCP solution in toluene (10 mg mL^{-1}) was pipetted on each surface to promote the formation of a self-assembled block copolymer monolayer at the air-water interface. After 5 min, the liquid level was raised slowly by injecting more salt solution below the surface, in turn raising the BCP monolayer above the hole level on both sides of the Teflon partition, thereby folding a bilayer membrane onto it (**Supplementary Figure 9**). Ag/AgCl electrodes were then inserted in each chamber to perform electric measurements for membrane characterization.

Osmotically matching DEX and PEO in salt solutions

To generate electric power from a transmembrane ion gradient, KCl was added to the DEX phase and NaCl to the PEO phase. To avoid the exertion of osmotic pressure on the membrane from one of the phases, we osmotically matched the DEX/KCl and PEO/NaCl solutions. 0.5 M KCl was dissolved in the 197 mg mL^{-1} DEX solution, resulting in an osmolality of 885 mOsm kg^{-1}. Different solutions were prepared to contain 0.2, 0.3, 0.4, 0.5 M NaCl, and 120 mg mL^{-1} PEO. The osmotic pressure of these solutions was measured and linear regression was used to determine the NaCl concentration (0.39 M) needed to match the osmotic pressure of the DEX/KCl phase (**Supplementary Figure 3)**

Preparation of the dry AFM samples

A piece of silicon wafer was cut with a glass cutting pen into a rectangle with around 2 mm width and 4 mm length. The silicon rectangle was rinsed with isopropyl alcohol and dried with nitrogen flow. A DEX solution was placed at the bottom of a vial, after which the wafer was dipped and held about ¾ of its length into the polymer solution using a linear positioner. A membrane was then formed using the toluene displacement method described in the main text. The piece of silicon was slowly pulled out of the solution carrying away part of the BCP membrane. The wafer was very gently washed with ultrapure water to remove excess PEO and DEX as well as salts before air drying.

Electric device with cells in series

Using FreeCAD software, a 3D model of the electric device was drawn in three parts (**Supplementary Figure 10**). The middle mold contains the channels that will support the BCP membranes, the bottom mold connects the channels in the horizontal direction while the top mold connects the cylinders in the vertical direction. Holes for screws were placed in all four corners of the device in order to tightly assemble the three molds. The design was 3D printed using a Formlabs Form 3+ stereolithography printer with Formlabs V4 Clear resin. In order to avoid leakage of electrolyte solution, a silicone elastomer gasket was made by curing Sylgard 184 according to the manufacturer's procedure in a large petri dish, to obtain a clear, flat sheet of silicone rubber with a thickness of approximately 1 mm. Then, two sheets of rubber were cut to the dimensions of the 3D-printed device (90x60 mm) and holes were applied in the positions of the channels and the screws. Silicone vacuum grease was used additionally to stick the rubber gaskets onto the device.

The channels of the device, with cross-sectional diameters of 5 mm, act as communicating vessels, which makes it impossible to form the membranes one by one. To circumvent this problem, the bottom mold was filled with a solution composed of 197 mg mL^{-1} DEX in 2 M KCl at a temperature $T > 55$ °C. After cooling, the solution turned into a gel thanks to a specific interactions between dextran and the potassium ions[4] isolating hydrostatically the channels while ensuring high electric conductivity. The device was then assembled and membranes were formed in every other channel with the toluene displaceement method using the DEX/KCl and PEO/NaCl solutions described above (see **Figure 4 E** in the main text). The outermost channels (**Supplementary Figure 10**) were used solely to introduce the Ag/AgCl electrodes and were both filled with DEX/0.5M KCl solution to avoid a potential offset because of differences in Cl^- concentration.

B. Supplementary Discussion

1. Thermodynamics of asymmetric membrane formation at the ATPS interface

The free energy of an aqueous two-phase system (ATPS) can be written as

$$F = F_0 + \gamma_{\mathrm{ATPS}} A \tag{SE1}$$

where γ_{ATPS} is the interfacial tension of the system, A is the contact area of the two phases and F_0 is a term containing all other contributions to the free energy. To study the thermodynamics of self-assembly at the ATPS interface, we first focus on the case where two different types of bilayer-forming block copolymers (BCP1 and BCP2) are assembled in the two phases (P1 and P2) of the ATPS, respectively. Both block copolymers will form vesicular structures in their respective phases because the formation of a spherical bilayer avoids the creation of energetically unfavorable edges.[5] We assume that BCP1 is immiscible in P2 and vice versa so that we can approximate the free energy of the system to

$$F_{\mathrm{A}} \cong F + N_{\mathrm{BCP1}} f_{\mathrm{BCP1}}^{\mathrm{P1}} + N_{\mathrm{BCP2}} f_{\mathrm{BCP2}}^{\mathrm{P2}} = F_0 + \gamma_{\mathrm{ATPS}} A + N_{\mathrm{BCP1}} f_{\mathrm{BCP1}}^{\mathrm{P1}} + N_{\mathrm{BCP2}} f_{\mathrm{BCP2}}^{\mathrm{P2}}. \tag{SE2}$$

The terms N_{BCP1} and N_{BCP2} designate the numbers of block copolymer molecules while $f_{\mathrm{BCP1}}^{\mathrm{P1}}$ and $f_{\mathrm{BCP2}}^{\mathrm{P2}}$ are the energies per block copolymer molecule in the vesicles. For the two block copolymers, the terms $f_{\mathrm{BCP}}^{\mathrm{P}}$ are functions of the stretching degree of the hydrophobic and hydrophilic blocks (entropic elasticity), of the interfacial tension between the hydrophobic blocks and water, of the cross-sectional area occupied by a block copolymer molecule in the bilayer (α) and of the enthalpic interaction between the hydrophilic block and the surrounding phase.[6]

Let's now imagine forming an asymmetric block copolymer bilayer at the interface between P1 and P2, where the bilayer side facing P1 is entirely composed of BCP1 and the side facing P2 is entirely made of BCP2. To totally cover the ATPS interface a certain number of copolymer molecules are needed, given by:

$$N_{\mathrm{BCP1}} = \frac{A}{\alpha_{\mathrm{BCP1}}}, \qquad N_{\mathrm{BCP2}} = \frac{A}{\alpha_{\mathrm{BCP2}}} \ . \tag{SE3}$$

The energy of such a system is given by

$$F_{\mathrm{B}} \cong F_0 + N_{\mathrm{BCP1}} g_{\mathrm{BCP1}}^{\mathrm{P1}} + N_{\mathrm{BCP2}} g_{\mathrm{BCP2}}^{\mathrm{P2}} + F_{\mathrm{E}}, \tag{SE4}$$

Where $g_{\mathrm{BCP1}}^{\mathrm{P1}}$ and $g_{\mathrm{BCP2}}^{\mathrm{P2}}$ are the energies per molecule of the block copolymers in the planar bilayer and F_{E} contains the excess free energy associated with the bilayer edge. The term $\gamma_{\mathrm{ATPS}} A$ present in **Supplementary Equation 2** disappears because the two phases are not in direct contact.

It is reasonable to assume that $f_{\mathrm{BCP}}^{\mathrm{P}} = g_{\mathrm{BCP}}^{\mathrm{P}}$ because the typical radius of block copolymer vesicles is much larger than the typical bilayer thickness, and therefore the BCP molecules do not feel the curvature. Hence, the energy difference between forming block copolymer vesicles in the two phases or assembling the block copolymers at the ATPS is:

$$\Delta F = F_{\mathrm{B}} - F_{\mathrm{A}} \cong -\gamma_{\mathrm{ATPS}} A + F_{\mathrm{E}} \tag{SE5}$$

For bilayers with areas approaching macroscopic areas, edge effects become negligible when compared to interfacial effects ($|\gamma_{\mathrm{ATPS}} A| \gg |F_{\mathrm{E}}|$). Hence, the energy gain upon forming a large-area self-assembled bilayer at the ATPS interface, as compared to the energy of forming dispersed vesicles is

$$\Delta F \approx -\gamma_{\mathrm{ATPS}} A \tag{SE6}$$

Thus, the formation of a 1 mm^2 bilayer at a PEO/DEX ATPS interface ($\gamma \approx 0.01\ k_\mathrm{B}T \cdot \mathrm{nm}^{-2}$) is associated with a $\Delta F \approx 10^{10}\ k_\mathrm{B}T$.

2. Control experiments

Membranes prepared with the folding method

We investigated the ability of PEO_{2kDa}-*b*-$PDMS_{5kDa}$ (BCP1) and $PDMAEMA_{2kDa}$-*b*-$PDMS_{5kDa}$ (BCP2) to form planar bilayers using the well-established folded planar bilayer method described above. This method uses a vertically placed poly(tetrafluoroethylene) film with a central hole to support bilayer formation.[7] The bilayer formed across the hole separates two electrolyte-filled chambers, allowing electric measurements with the aid of Ag/AgCl electrodes placed in the two compartments. Applying time-varying potentials enables to measure the electric capacitance (and hence the thickness d) and the specific resistance R_SP of the bilayer if its area A is known. These experiments (**Extended Data Figures 1**) reveal that the two block copolymers form stable membranes (both when used individually or mixed in equal weight proportions) with specific resistance of $0.1 < R_\mathrm{SP} < 10\ \mathrm{M\Omega \cdot cm^2}$, comparable to the specific resistance range of lipid bilayers.[8–10] The average bilayer thickness varied between $8 < d < 20$ nm, presumably depending on the entrapment of small amounts of toluene.

The two block copolymers were expected to form membranes with similar properties as they share the same hydrophobic PDMS blocks and have hydrophilic blocks with similar lengths. However, when using only PDMS-*b*-PDMAEMA, having the shorter water-soluble stabilizing block, it was not possible to form stable membranes across the 500 µm hole and the temporal stability (time-to-break) of the smaller membranes was lower than that of the membranes using only the PEO counterpart or both polymers.

Control experiments with the ATPS

To check whether stable membranes are formed at the ATPS interface because of the preferential positioning of the two different hydrophilic blocks in the non-mixing phases, control experiments were performed in triplicate, using 1) DEX on both sides, 2) PEO on both sides, 3) PDMAEMA-*b*-PDMS only and 4) PEO-*b*-PDMS only. None of the above situations yielded significantly stable membranes ($t_\mathrm{life} > 10$ s), consisting in thin bilayers ($d < 100$ nm) and with high resistance ($R > 1$ MΩ). The mean resistance values from three independent samples for each situation are shown in **Supplementary Figure 11**, demonstrating no bilayer membranes were formed.

Membranes with PHMA as hydrophobic blocks

To demonstrate that the toluene displacement method is general and not limited to PDMS-based BCPs, another set of BCPs with poly(hexyl methacrylate) (PHMA) hydrophobic block was prepared and used. A mixture of $PHMA_9$-*b*-PEO_{43} and $PHMA_9$-*b*-$PDMAEMA_9$ (1:1 by weight) was dissolved at a total concentration of 50 mg mL^{-1} in toluene. Then, the membranes were assembled using the ATPS as support as described in the Methods section in the main text. For these experiments, we used cylindrical cells with a cross-sectional diameter of 4.5 mm. The observed membrane properties (R_SP, d, and the specific capacitance C_SP) were similar to those obtained using PDMS-containing block copolymers (**Supplementary Table 1** and **Supplementary Figure 12**), confirming that the ATPS approach can be extended to various types of BCP membranes.

Control experiment with a peptide that is not an ionophore

To demonstrate that shuttling of ions by valinomycin (VM) is the mechanism behind the observed increase in transmembrane current upon addition of the peptide, a control experiment was performed

using a non-ionophoric peptide with a comparable molar mass. We chose Alamethicin (AM) since this peptide also resides in the cell membrane[11]. I-V curves (**Supplementary Figure 13**) comparing the same molar concentration of either VM or AM (2 mol% peptide in the BCP solution in toluene) in a planar bilayer membrane. Incorporation of VM results in a substantial increase in the magnitude of the transmembrane current and the appearance of an offset potential, whereas the resistivity remains high and no offset potential is observed after incorporation of AM, as expected.

3. Characterization of the membranes formed at the ATPS interface

Evolution over time and thickness

The addition of salt (NaCl) to the ATPS system enables electric measurements that allow monitoring the formation of these ATPS-stabilized membranes over time. In the initial stage, before the bilayer formation is complete, the average membrane thickness is on the order of $d \approx 300$ nm (**Supplementary Figure 14**), indicating that the two BCP monolayers entrap a relatively thick layer of organic solvent. Over time, the solvent is expelled from the membrane, whose thickness decreases over the course of 10-50 min (depending on the experiment) to a median thickness of $d \cong 35$ nm and interquartile range of 28 nm independent of the membrane area (**Figure 2A** in the main text). This average thickness is about twice as high as the one measured for membranes produced with the bilayer folding method, suggesting that a significant amount of toluene remains entrapped in the membranes after 1 h. To provide an independent, direct measurement of the bilayer thickness and exclude the formation of multilayered membranes, fragments of an ATPS-stabilized membrane were transferred onto a silicon wafer, dried, and characterized via atomic force microscopy (AFM, details in Supplementary Methods). The analysis of the AFM micrographs (**Supplementary Figure 15**) shows that the membrane thickness is homogeneous and has an average value of 33 ± 3 nm. Note that this estimate also includes a contribution from the hydrophilic blocks and that therefore the actual thickness of the hydrophobic core is smaller, indicating that the residual toluene left the membrane during drying. This observation, combined with the absence of multi-layers, corroborates that the large-area membranes are self-assembled bilayers and that their thickness is slightly increased by the entrapment of toluene. The holes present in the membrane that can be seen in the AFM image formed during the preparation of the AFM sample. In fact, the high resistance (>1MΩ) prior sample preparation indicates that no holes were present in the hydrated membrane. Coarse-grained molecular dynamics (CGMD) support this hypothesis by showing that holes, similar to those observed by AFM, form when the membrane is subject to a stress (see **Supplementary Discussion 4**).

Reproducibility

We used the solvent displacement method to prepare more than a hundred ATPS-stabilized membranes and observed the formation of stable membranes with thicknesses below 100 nm and specific resistance larger than 10^4 Ω·cm^2 in 80% of the experiments performed. Failure in the remaining 20% of the cases was probably due to the formation of BCP monolayers with defects or to instantaneous rupture caused by interfacial stresses arising during the addition of the PEG phase.

Surface area and stability

We varied the cross-section of round vessels in which the membranes were produced, and were able to assemble membranes with a diameter of up to 1.3 cm, i.e. an area of up to $A = 1.4\ \mathrm{cm}^2$, above which membranes started to break after a few minutes. Fixing one of the dimensions to 0.5 cm and extending the membranes perpendicularly made it possible to surpass this limit. To this end, we designed and 3D printed elongated polymeric cells with slanted floors that enable the control of the membrane area by varying the volume of the two phases (**Extended Data Figure 4 A-C**). With these cells, we produced

membranes with areas up to $A = 8.75\ \text{cm}^2$. Finally, we designed and 3D-printed containers with supporting pillars (**Extended Data Figure 4 D**) that enabled the formation of membranes as large as $A = 10\ \text{cm}^2$, which is three orders of magnitude larger than the largest self-assembled membrane produced with the folding method[12,13] and, to our knowledge, by far the largest free-standing self-assembled bilayer membrane reported to date. The large-area membranes were, in fact, typically stable for several hours, wherein with stability we mean that the specific resistance of the membrane remained larger than $10^4\ \Omega\cdot\text{cm}^2$ and the thickness larger than 10 nm.

Permeability

Figure 2A in the main text shows that the specific resistance of large-area ATPS-stabilized membranes consistently exceeds $0.1\ \text{M}\Omega\cdot\text{cm}^2$. Hence, despite the large area and an average thickness below 40 nm, ATPS-supported membranes possess excellent barrier functions that are comparable to lipid membranes. These interface-supported planar copolymer membranes are not only impervious to ions, but also for charged water-soluble organic molecules, as confirmed by leakage tests shown in **Figure 3A** in the main text and **Supplementary Figure 16**. After forming a membrane, a fluorophore such as calcein (**Figure 3A**) was added to the top (PEO) phase. Within two hours, the dye diffused homogeneously in the PEO phase but did not cross the membrane, in contrast to the control experiment with an ATPS but without a membrane formed at the interface. In a separate experiment, three different types of dyes were selected that are soluble in the aqueous PEO solution: a repeat experiment with calcein (anionic) and new experiments with methylene blue (cationic), and rhodamine B (amphoteric). From a 1 mg mL^{-1} stock solution in water, they were diluted in the PEO solution to reach 1 μg mL^{-1} final concentration. Then, the membranes were assembled at the ATPS interface as described before, with the respective dyed PEO solutions on the top. Membrane formation was confirmed by measuring a resistance of the 4 mm wide membranes with $R > 1\ \text{M}\Omega$ in all cases. Then, the membranes were left, and photos were taken at the start, after 4, and 8 hours (**Supplementary Figure 16**). The dyes did not cross the membrane in the first hours, however, after 8 hours, the methylene blue was visible in the bottom phase (the resistance of the membrane was $R < 10\ \text{k}\Omega$, meaning this membrane had broken, allowing dye to pass)

Mechanical properties

To evaluate the mechanical resistance of the ATPS-supported membranes, we purposedly created an osmotic pressure mismatch between the DEX and the PEO phases via addition of NaCl in the PEO phase. Stable membranes formed up to an osmotic pressure difference of approximately 4 kPa (a difference in NaCl concentration of approximately 4 M), after which we observed the formation of membranes with defects, as testified by a low (<1MΩ) resistance (**Supplementary Figure 17**). Above an osmotic pressure mismatch of 6 kPa we did not observe membrane formation (**Supplementary Figure 17**). This experiment provides an estimate of the mechanical stability of the ATPS-supported membranes, that can withstand osmotic pressure differences of approximately 4 kPa.

We also measured the penetration force of the ATPS-supported membranes via AFM. **Figure 2D** in the main text shows the cantilever force versus tip-sample distance of an ATPS-supported membrane in its native, hydrated state. The abrupt change on the red force curve at 0.3 μm distance represent the penetration of the membrane, from which we obtained a penetration force of 4.3 nN. The darker regions on the membrane plane represent small variations in the membrane thickness of approximately 6 nm (**Supplementary Figure 1**), much smaller than the total membrane thickness (~40 nm) determined via capacitive measurements.

Membrane asymmetry

We used Förster resonance energy transfer (FRET) to verify whether the membranes that form at the interface of aqueous two-phase systems are asymmetric or not. The FRET process takes place when a donor molecule is excited by light and transfers the gained energy to an acceptor dye. Subsequently, the acceptor dissipates the transferred energy by emitting a photon within a specific wavelength range, different from the emission range of the donor[13]. The choice of the donor-acceptor couple is very important as it determines the efficiency of the FRET process. Ideally, the absorption spectrum of the donor should be well separated from the absorption spectrum of the acceptor while the emission spectrum of the donor should have a large overlap with the absorption spectrum of the acceptor. These spectral features make it possible to selectively excite the donor (without directly exciting the acceptor) and record the FRET emission of the acceptor.

The asymmetry of the membrane can be probed by labelling one of the two membrane-forming block copolymers with a FRET donor and measuring the FRET emission of an acceptor when this is dispersed only in the PEO or only in the DEX phase (**Supplementary Figure 18 A**). The FRET process takes place if the distance between donor and acceptor is smaller, typically, than 10 nm[13]. Since the thickness of the membrane is much larger (>30 nm) than this critical distance, the acceptor molecules at one side of the membrane can be excited only by donor-labelled block copolymers that are in that side of the bilayer. Hence, if the membrane is compositionally symmetric, adding the acceptor molecule at either side will result in similar FRET intensities. Conversely, an asymmetric membrane will yield different FRET intensities depending on where the acceptor is added (**Supplementary Figure 18 A**).

We selected the well-established donor-acceptor FRET couple Cyanine 3 (Cy3, donor) and Cyanine 5 (Cy5, acceptor) because both dyes are water-soluble. We synthesized a PEO-PDMS block copolymer labelled with Cy3 as described in the Supplementary Methods section. Using a custom-made microscopy setup (**Supplementary Figure 18 B**), we were able to form ATPS-supported membranes on an inverted confocal microscope while performing fluorescence and dielectric measurements simultaneously. We used the dielectric measurements to monitor the formation of the membrane, that is difficult to resolve via fluorescence microscopy due to small thickness of the membranes. We used a PicoQuant MicroTime 200 equipped with a Solea pulsed white laser, a 10x Olympus plan N objective and a 30 µm pinhole to perform the measurements. We selected an excitation wavelength of 510 nm, and we pre-filtered the emission signal with a 532 nm long-pass filter. We split the filtered emission signal in two detection bands, 655-725 nm (Channel 1) and 532-575 nm (Channel 2), by means of a 635 nm dichroic and appropriate band-pass filters placed in front of two SPAD detectors. Channel 1 mostly contains the emission signal from Cy5 while Channel 2 contains mostly the signal from Cy3 (**Supplementary Figure 18 C-E**).

We formed membranes with the method described in the main text using a BCP solution containing 25 mg ml^{-1} of BCP2, 24 mg ml^{-1} of BCP1 and 1 mg ml^{-1} Cy3-labelled PEO copolymer in toluene. To perform the FRET experiments, we dissolved Cy5 at a concentration of 5 µg ml^{-1} directly into either the PEO or the DEX phase prior to membrane formation. In all experiments, we used the same laser power and we adjusted the focus of the microscope maximizing the fluorescence intensity in Channel 2, then we waited for the membranes to reach a capacitance of 7 nF to record the time-correlated single-photon counting (TCSPC) histograms of Channel 1 and Channel 2. We fitted the resulting decay curves (**Supplementary Figure 18 C-G**) with a bi-exponential function, which provided a good fit in all cases:

$$f(t) = A_1 e^{-\frac{t}{\tau_1}} + A_2 e^{-\frac{t}{\tau_2}} + B. \quad \text{(SE7)}$$

The term B is a constant that describes the background noise. Provided that the repetition time t_{R} of the laser is long enough to sample the entire decay (i.e. $f(t_{\mathrm{R}}) \approx B$), the total number of photons I_{T} recorded can be written as:

$$I_{\mathrm{T}} = \int_0^{t_{\mathrm{R}}} f(t)dt \approx A_1\tau_1 + A_2\tau_2 + Bt_{\mathrm{R}}. \quad \text{(SE8)}$$

Supplementary Equation 8 makes it possible to discern the integrated fluorescence intensity $I_{\mathrm{F}} \approx A_1\tau_1 + A_2\tau_2$ from the integrated background noise $I_B = Bt_{\mathrm{M}}$. The ratio (R_{FRET}) between the I_{F} values obtained from Channel 1 ($I_{\mathrm{F}}^{\mathrm{Ch1}}$) and Channel 2 ($I_{\mathrm{F}}^{\mathrm{Ch2}}$) is linked to the efficiency of the FRET process, hence it provides a way to assess the asymmetry of the membrane qualitatively:

$$R_{\mathrm{FRET}} = \frac{I_{\mathrm{F}}^{\mathrm{Ch1}}}{I_{\mathrm{F}}^{\mathrm{Ch2}}} = \frac{A_1^{\mathrm{Ch1}}\tau_1^{\mathrm{Ch1}} + A_2^{\mathrm{Ch1}}\tau_2^{\mathrm{Ch1}}}{A_1^{\mathrm{Ch2}}\tau_1^{\mathrm{Ch2}} + A_2^{\mathrm{Ch2}}\tau_2^{\mathrm{Ch2}}}. \quad \text{(SE9)}$$

Supplementary Table 2 lists the I_{F} and R_{FRET} values obtained in the FRET experiments as well as three control experiments (a Cy3-labelled membrane with no Cy5, Cy5 only in the aqueous DEX phase and Cy5 only in the aqueous PEO phase). As it can be seen, the addition of Cy5 on either side of the membrane causes an increase in R_{FRET} as compared to the bare membrane case. The R_{FRET} value obtained when Cy5 is added on the PEO phase is twice that obtained when Cy5 is added on the DEX side. This difference indicates that the Cy3-labelled PEO block copolymer is present to a larger extent on the PEO side than on the DEX side, demonstrating that the membrane is, to some extent, asymmetric.

A further proof of the membrane asymmetry comes from an analysis of the mean fluorescence lifetimes, defined as:

$$\tau_{\mathrm{m}} = \frac{A_1\tau_1 + A_2\tau_2}{A_1 + A_2}. \quad \text{(SE10)}$$

It is known that with increasing the extent of the FRET process, the fluorescence lifetime of the donor decreases because long-living excited states are more probable to transfer energy than short-living states. Conversely, the mean lifetime of the acceptor increases because the time it takes from the excitation of the donor to the energy transfer sums to the lifetime of the excited state of the acceptor. When Cy5 is dispersed in the PEO phase, we register the shortest lifetime of the donor and the longest lifetime of the acceptor (**Supplementary Table 3**) indicating that the PEO side of the membrane is richer in PEO block copolymers than the DEX side, in agreement with the intensity measurements.

4. Coarse-grained (CG) molecular dynamics simulations

The generic modelling strategy employed describes all polymers as simple bead-spring chains, which capture the essential properties of linear polymers. Both the bulk DEX and PEO phases consist of long chains of length 200 CG beads, which is sufficiently long to allow the formation of entanglements. Bonded interactions between segments include both stretching and bending valence terms. Bonded (stretching) interactions between nearest neighbouring beads along the polymer chains are captured via a harmonic spring potential

$$U_{\mathrm{bond}} = k_{\mathrm{bond}}(l - l_0)^2 \quad (SE11)$$

which describes the potential energy change associated with deforming the polymer bonds away from the equilibrium separation $l_0 = 0.967$. Note this value is chosen to prevent unphysical bond crossings in all species, see **Supplementary Table 4**. The rigidity of the different polymer species is enforced using simple harmonic angular potentials of the form

$$U_{\text{angle}} = k_{\text{angle}} (\theta - \theta_0)^2 \quad (SE12)$$

and describes the potential energy change associated with bending the angle θ formed by 3 nearest neighbouring beads from the equilibrium angle of $\theta_0 = 180°$, i.e. a perfectly straight polymer chain. The higher persistence length of DEX compared to PEO is captured by a doubling of the constant k_{angle}, see **Supplementary Table 4**. Non-bonded interactions take place via a standard Lennard-Jones (LJ) potential of the form

$$U_{LJ} = 4\varepsilon_{AB}\left(\left(\frac{\sigma}{r}\right)^{12} - \left(\frac{\sigma}{r}\right)^{6}\right), \quad r < r_c \quad (SE13)$$

where ε_{AB} is the depth of the potential well and σ the zero-crossing or particle size and r is the separation between particles. The potential is cut and shifted to zero at the cut-off $r_c = 2.3\ \sigma$ ensuring some of the attractive tail of the LJ potential is incorporated. Immiscibility between different species is enforced through the effective ratios of their potential well depths. The potential parameters set the length and energy scales of the simulation; the particle size is set to $\sigma = 1$ nm for all species.

The mixing parameters ε_{AB} between different polymers are first estimated using an elegant method described in[14,15] which relates the theoretical critical point of the liquid-gas of the real polymer to that of a LJ fluid using experimental data[16]. The model used is quantitatively identical to ours, apart from the additional angular potentials which impart additional rigidity to the polymer. This calculation is known to be approximate but nonetheless serves as a starting set of parameters, from which a stable bilayer can be obtained with subsequent tuning. From this calculation, it was found that $\varepsilon_{PEO-PEO} \approx \varepsilon_{DEX-DEX} \approx 2\ \varepsilon_{DMS-DMS} \approx 2\varepsilon_{H_2O-H_2O}$, thus we scale all parameters such that $\varepsilon_{\ PEO-PEO} = \varepsilon_{DEX-DEX} = 1$, see **Supplementary Table 5.**

Mixing terms between species are obtained using a variant of the Berthelot rule

$$\varepsilon_{AB} = \xi_{AB}\sqrt{\varepsilon_{AA}\varepsilon_{BB}} \quad (SE14)$$

where $\xi = 1$ in the standard rule. The value ξ has a profound effect on the phase diagram, for $\xi > 1$ the different species A or B attract each other more than they attract themselves, whereas for $\xi < 1$ each species attracts itself more than it attracts the other, leading to phase-separation[17]. The tuned values which support a stable bilayer under equilibrium conditions are as follows:

$$\xi_{PEO-H_2O} = \xi_{DEX-H_2O} = 1.131$$

$$\xi_{DMS-H_2O} = 0.2$$

$$\xi_{PEO-DMS} = \xi_{DEX-DMS} = 0.566$$

$$\xi_{PEO-DEX} = 0.9$$

The critical parameters to stabilise the bilayer were primarily the hydrophobicity of the PDMS tails and the repulsion between head and tail species of the BCPs. The masses of all beads are set to 1.0.

Methodology

Equilibration and production simulations are performed using LAMMPS[18,19]. The polymer melt (PEO-DEX) was obtained by populating a cubic box of size 65σ with 600 elongated chains of each species, with length 200 beads, partitioned on either side of the simulation cell to promote the formation of an

interface under NVT conditions. Equations of motion are integrated using a velocity-Verlet algorithm with a timestep of 0.005 τ, and temperature is held fixed at 1 $k_B T$ using a Langevin thermostat with coupling constant 2.0 τ. The polymer melt is equilibrated for 100 $k\tau$, and metrics commonly used to characterize well equilibrated systems of polymers, including the internal distance distribution and end-to-end vector auto-correlation function, are continuously monitored. Simulations are quoted in terms of reduced LJ units such that quantities are normalised by σ and ε and the characteristic time $\tau = \sqrt{m\sigma^2/\varepsilon}$.

Once the bulk polymer phases have been obtained with a stable interface, 662,400 solvent particles and 9917 BCP molecules of length 23 beads are forced into the polymer melt using hybrid potentials, where interactions with BCPs and solvent beads take place via a soft potential of the form $U_{\text{soft}} = A\left[1 + \cos\left(\frac{\pi r}{r_c}\right)\right]$ at short times. This ensures no infinite forces as the particles are pushed out into the melt. The system is then allowed to relax for 50 τ before switching back to the full LJ potential with the interaction parameters specified in **Supplementary Table 5**. Such a short time is used to ensure the particles are simply pushed out before drifting too far away from their initial positions. The equilibrium pressure of 6.5 $\varepsilon\sigma^{-3}$ is applied using an anisotropic Nose-Hoover barostat, coupled in x,y, with a time constant of 100 τ. This ensures the reduced density $\rho^* \approx 1$. A small resorting force is applied to the BCP head groups to drag them to one side of the interface such that the bilayer begins in an asymmetric configuration (**Supplementary Figure 19**) and is subsequently removed after 5 $k\tau$.

Production runs of the stable bilayer were equilibrated for 2 Mτ and equilibration identified by a plateau in box dimensions and a stabilization of BCP partitioning across the interface. A series of snapshots of the fully equilibrated double bi-layer system, with different components, is shown in **Supplementary Figure 20**.

Concentration Profiles

Undulations in the bilayer are removed to obtain precise concentration profiles by first binning all species into cubic bins of length ~$2\sigma^3$ across the simulation cell and locating the center of the bilayer. The center of the bilayer is assumed to correspond to the bin with the highest volume fraction of the PDMS component for a given slab of bins in the z direction. The first slab serves as a reference, and all subsequent slabs are shifted to ensure their respective centers are aligned. This results in a series of concentration profiles, one for each slab, which are then averaged and smoothed. An example of undulation-corrected concentration profile is displayed in **Figure 2** in the main text. For reference, the concentration profile obtained without this procedure is plotted in **Supplementary Figure 21**, which shows significant broadening due to bilayer undulations. Consistent with both the FRET measurements and SCF calculations, the simulations reveal that the bilayer is asymmetric. The initial perfectly asymmetric starting configuration turns progressively asymmetric, until it settles at approximately 70%. It was observed that the transport of BCPs between the respective sides of the bilayer takes place through the formation of small holes. In this way a BCP may diffuse along the interface, to the opposite side, without incurring a penalty for pulling the hydrophilic group through the hydrophobic part of the bilayer. A small fraction of solvent was also noted to remain trapped within the hydrophobic tails of the BCP bilayer. The equilibrium interfacial width is also remarkably close to the experimentally observed 30 nm in AFM.

Mean-Squared Displacement and Diffusion

The mean-squared displacement (MSD) of the monomer particles is defined by

$$g_0(t) = \frac{1}{N}\sum_{i=1}^{N}\langle|\boldsymbol{r}_i(t) - \boldsymbol{r}_i(0)|^2\rangle \qquad (SE15)$$

where $\boldsymbol{r}_i(t)$ is the position of the particle i at time t and the sum runs over all (N) BCP monomers. The MSD of the centre-of-mass (COM) of the chain is similarly given by

$$g_{CM}(t) = \frac{1}{M}\sum_{i=1}^{M}\left\langle\left|\boldsymbol{r}_{CM_i}(t) - \boldsymbol{r}_{CM_i}(0)\right|^2\right\rangle \qquad (SE16)$$

where $\boldsymbol{r}_{CM_i}(t)$ is the position of the COM of a given chain at time t and the sum runs over all (M) BCP chains. Note both definitions should superpose perfectly at long times in the diffusive regime. The diffusion coefficient can be calculated from the slope of the MSD where $g_0(t) = 6Dt$ with D being the time dependent diffusion coefficient of the BCP chains (**Supplementary Figure 22**). We note the diffusion coefficient is effectively 2D in the long-time limit i.e. $\lim_{t\to\infty} g_0(t)/t = \lim_{t\to\infty} g_{CM}(t)/t = 4D$ since $g_0^{(z)}(t) \to 0$ for large t.

In order to obtain a reasonable estimate of the BCP translational diffusion coefficient we renormalize time in units of the typical lateral Brownian diffusion time per bead instead of the standard MD time $\tau = \sqrt{m\sigma^2/\varepsilon_{PEO-PEO}}$. The Brownian timescale, defined as $t_{diff} = a^2/4D_b$, is the typical time a bead with radius a needs to diffuse over a distance comparable to its size in a frictional solvent (water). It is determined by the Stokes-Einstein diffusion coefficient $D_b = k_BT/6\pi\eta a$ in terms of the shear viscosity of water $\eta = 10^{-3}Pa.s$. Taking the average mass of a BCP bead to be approximately m =232 Da and $\varepsilon_{PEO-PEO} = 1.30 \times 10^{-20}$J and performing the time conversion we find that the diffusion coefficient of a BCP within the bilayer $\boldsymbol{D} \approx \mathbf{8}$ μm^2/s which is within the typical expected range $\boldsymbol{D} \approx \mathbf{2-10}$ μm^2/s for lipid bilayers

Self-Healing

Once the equilibrium bilayer partitioning has been obtained, a biaxial deformation is performed where the anisotropic barostat is decoupled in the x and y directions and the box is stretched at a constant true strain rate of 10^{-4} τ^{-1}. This is performed until a series of substantial holes appear in the bilayer. Once the holes are sufficiently large, the deformation is halted, the barostat is recoupled in the x,y-direction and self-healing is allowed to take place. See **Supplementary Video 1**. Self-healing of the BCP bilayer structure was also observed in our coarse-grained molecular dynamics simulations. Here the bilayer is stretched biaxially in the x-y direction (**Supplementary Figure 23**) at a constant true strain rate of 10^{-4} τ^{-1} until large holes begin to appear in the bilayer, after which the deformation is stopped and the system is allowed to relax at constant pressure, temperature and number of molecules. **Supplementary Figure 24** shows a series of snapshots at different times during the self-healing procedure.

The Swiss-cheese like structure of the bilayer under biaxial deformation resembles the AFM images in **Supplementary Figure 15**. This suggests that stresses induced during sample preparation (which included multiple washing cycles and drying in the case of these particular AFM studies) may be responsible for the large holes observed in the AFM micrographs of the bilayer. Such a strong correspondence between experiment and simulation suggests that this phenomenon can be generalised to other self-assembled bilayers.

5. Self-consistent field studies of ATPS-supported membranes

We used self-consistent field (SCF) computations to investigate theoretically the formation of block copolymer membranes at the interface of an aqueous two-phase system. We used the well-established

Scheutjens-Fleer SCF (SF-SCF) theory, that has been extensively employed to study block copolymer self-assembly and is described in detail in the literature[20–22]. In brief, the SF-SCF theory is based on Flory-Huggins theory[23] and considers concentration gradients and inhomogeneities by solving Edwards diffusion equations. Enthalpic interactions between pairs of components are modelled by means of Flory-Huggins interaction parameters (χ_{ij})[23] and electrostatic interactions can also be considered.[22]

Taking advantage of the symmetry properties of our system, we solve the SF-SCF equations on a lattice considering concentration gradients along a single direction (1D-SF-SCF). The lattice consists of a collection of 100 lattice layers to which we assign, unless otherwise specified, a thickness of 1 nm. Concentrations are homogeneous within the same layer but they can vary between different layers. The lattice is filled with the following components:

- 10% of the lattice is occupied by a polymer A_{200}, that mimics PEO
- 10% of the lattice is occupied by a polymer B_{200}, that mimics DEX
- a variable fraction of the lattice, ϕ, is occupied by block copolymers of the type A_n-C_m, mimicking the PEO-based block copolymers such as PEO-PDMS or PEO-PHMA
- the same fraction of the lattice ϕ is occupied by block copolymers of the type D_n-C_m, mimicking block copolymers that are compatible for the DEX phase, such as PDMAEMA-PDMS or PDMAEMA-PHMA.
- the remaining fraction of the polymer is filled with solvent molecules S (i.e. water).

Although the PEG and DEX polymers used in this work are quite different from each other in molar mass, dispersity and flexibility, we chose their SCF analogues to be equal in chain length and monodisperse because under our experimental conditions long (>100 monomers) polymer chains overlap and entangle, hence their behavior becomes only weakly dependent on the chain length.[24] The two block copolymers have also equal structures, and the chain length of their blocks is varied to explore how this parameter affects the properties of the membranes.

Since 5 different types of components are present (A, B, C, D, S), there are 10 pairwise interaction parameters that need to be specified. As the goal of these computations is to provide general insights on the formation of membranes at the interface of two immiscible polymer solutions, we used the effective interaction parameters listed and motivated in **Supplementary Table 6**.

To speed up convergence, the computations are initialized assuming that all A_{200} polymers are confined in the first 45 layers and the B_{200} polymers are confined in the last 45 layers. The block copolymer molecules are instead confined in the central 10 layers (from 45 to 55). After an initialization run, the constraints are removed, and the energy of the system is minimized.

Membrane asymmetry

We first performed a computation in the absence of block copolymers ($\phi = 0$), and we verified that a stable interface formed at the center of the lattice. Then, the SCF free energy is minimized for increasing ϕ values (i.e. adding a growing number of block copolymer molecules in the lattice) until the grand potential Ω reaches 0. The SF-SCF grand potential provides a measure of the energy penalty associated with the creation of interfaces, therefore at $\Omega = 0$ the bilayer is in its equilibrium configuration. The SCF machinery generates concentration profiles like the ones plotted in **Supplementary Figure 25**, which describe how the various components distribute in space at equilibrium in the direction perpendicular to the bilayer plane.

We selected block copolymers with compositions A_{20}-C_{40} and D_{20}-C_{40}, and we simulated the formation of a bilayer assuming that the A and D hydrophilic blocks interact only via hard-core interactions ($\chi_{AD} = 0$) and that A and B are weakly repulsive ($\chi_{AB} = 0.5$). In these conditions, a stable and quasi-symmetric self-assembled bilayer forms at the interface between the A and B solutions (**Supplementary Figure 25**). Notably, the hydrophilic A blocks facing the B-rich phase are less stretched than the hydrophilic D

blocks (**Supplementary Figure 25 A**, see region marked by a green square). This result suggests that a symmetric bilayer can form despite the repulsive interaction between A and B because the D blocks, compatible with the B polymers, screen the A blocks from the unfavorable contact with B_{200}. This picture becomes even more evident if the repulsion between A and B is increased ($\chi_{AB} = 1$, **Supplementary Figure 25 B**).

When a repulsive interaction between the A and D blocks is introduced ($\chi_{AD} > 0$), the bilayer becomes progressively asymmetric (**Supplementary Figure 25 B and C**), with the A and D blocks accumulating at opposite sides of the bilayer. In **Supplementary Figure 26 A** we plotted the percentage of A_{20}-C_{40} block copolymers at the side of the bilayer facing the A_{200}-rich phase with respect to the total number of A_{20}-C_{40} block copolymer present in the bilayer. The plot shows that for $\chi_{AD} > 0.3$ the bilayer becomes largely asymmetric until it becomes almost totally asymmetric when $\chi_{AD} = 0.5$.

Supplementary Figure 26 B displays the degree of asymmetry of bilayers made of block copolymers with composition A_n-C_{2n} and D_n-C_{2n} when n is varied from 10 to 40 while χ_{AD} is fixed to $\chi_{AD} = 0.2$. The asymmetry of the membrane is strongly affected by the length of the block copolymers, especially of the hydrophilic blocks because the tendency of short (<100 units) polymers to demix increases strongly with increasing the chain length. Thus, the degree of asymmetry of the bilayer is governed by both the interaction between the hydrophilic blocks and their chain length.

To estimate the degree of asymmetry of the block copolymer membranes prepared in this work, we calculated the interaction parameter between PEO and PDMAEMA using the method proposed by Lidvig and coworkers[25]. The Hansen solubility parameters of PEO ($\delta_D = 16.3$, $\delta_P = 10.2$ and $\delta_H = 11.4$) were taken from the literature[26] while the parameters for PDMAEMA ($\delta_D = 17.4$, $\delta_P = 10.4$ and $\delta_H = 7.4$) were estimated with the group contribution method by Stefanis and Panayiotou[27]. We obtained an estimate of $\chi_{AD} = 0.12$. We also calculated the SCF-equivalent chain length of the two block copolymers employed to form the membranes. Using the PEO monomer as a reference and considering the mass ratio between the hydrophilic and hydrophobic blocks, we model the two block copolymers as A_{40}-C_{90} and D_{40}-C_{90} and we assign to each lattice layer a size of 0.4 nm.[26] With these parameters, the SF-SCF computations predict that approximately 70% of the total PEO-based block copolymers face the PEO phase in striking agreement with the MD simulations, hence the membrane is asymmetric.

Membrane thickness

We systematically varied the composition of the block copolymers and we studied how this parameter affects the thickness of the hydrophobic core of the bilayer, that is the domain that determines most of the transport properties of the membrane. As expected, we found that the thickness of the membrane scales linearly with the mass of the block copolymers if the ratio between the hydrophilic and hydrophobic blocks is held constant (**Supplementary Figure 26 C**). On the contrary, increasing the hydrophilic block length while keeping the hydrophobic block length constant leads to a linear decrease of the thickness of the hydrophobic core (**Supplementary Figure 26 D**). This is readily explained by theoretical models of block copolymer self-assembly[6]: the thickness of the hydrophobic core depends on how much the hydrophobic chains stretch. The degree of stretching is affected by how close the block copolymers are in the bilayer. Increasing the length of the hydrophilic blocks causes an increase in the steric repulsion between adjacent block copolymer molecules pushing them apart. This is associated with a relaxation of the hydrophobic blocks, leading to a decrease in the thickness of the hydrophobic core of the bilayer.

6. Viscosity requirements of the solvent displacement method

We applied the solvent displacement method introduced here to form membranes with various organic solvents, including ethyl acetate, toluene, m-xylene, p-xylene, and o-xylene. Stable membranes were formed with toluene, m-xylene, p-xylene and o-xylene, solvents which share a similar chemical structure and physic-chemical properties (**Supplementary Figure 27**). Since in theory the chemical structure of the solvent should not play a role in the assembly process[6], and since the self-assembly of block copolymer is known to be strongly dependent on kinetics[28], we hypothesized that there are certain viscosity requirements to form stable membranes. According to our understanding of the assembly process, the formation of ATPS-stabilized membranes is governed by two timescales, which are i) the rearrangement time of the block copolymer at the solvent-water interface and ii) the sinking/sedimentation time of the PEO phase, i.e. the time it takes for the PEO phase to cross the organic layer and reach the DEX interface. To form a stable membrane, the block copolymer molecules dispersed in the organic phase must form a homogeneous monolayer at the PEO interface before the PEO phase reaches the DEX interface. A possible explanation for the observed dependence on the solvent viscosity is that if the viscosity of the organic solvent is too low, the sinking time of the PEO phase is too short to allow the formation of a complete monolayer, while for larger viscosities (approximately $\eta > 0.5$ mPa·s according to our experiments) the sinking time is large enough to permit the formation of a stable monolayer at the PEO interface. To demonstrate this hypothesis, we added 25% (v/v) of n-decane, a high-viscosity solvent, to ethyl acetate to form a solvent mixture with viscosity $\eta \approx 0.7$ mPa·s. As shown in **Supplementary Figure 27**, after addition of 25% decane we were able to form stable membranes with ethyl acetate, while pure ethyl acetate did not yield stable bilayers, corroborating our hypothesis that viscosity plays a major role in the formation of ATPS-supported membranes.

7. Membrane permselectivity ratio and device power output.

The theoretically expected transmembrane potential (V_m) can be calculated using the Goldman-Hodgkin-Katz equation[29]:

$$V_{\mathrm{m}} = \frac{RT}{F}\ln\left(\frac{p_{\mathrm{K}}[\mathrm{K}^+]_{\mathrm{DEX}} + p_{\mathrm{Na}}[\mathrm{Na}^+]_{\mathrm{DEX}} + p_{\mathrm{Cl}}[\mathrm{Cl}^-]_{\mathrm{PEG}}}{p_{\mathrm{K}}[\mathrm{K}^+]_{\mathrm{PEG}} + p_{\mathrm{Na}}[\mathrm{Na}^+]_{\mathrm{PEG}} + p_{\mathrm{Cl}}[\mathrm{Cl}^-]_{\mathrm{DEX}}}\right) \quad \text{(SE17)}$$

Where $p_{\mathrm{K/Na/Cl}}$ are the relative membrane permselectivities for the respective ions, [ion] their concentrations, R is the universal gas constant, T is the temperature and F is the Faraday constant. **Supplementary Equation 17** can be simplified by assuming $p_{\mathrm{Cl}} \cong 0$. Under this assumption, justified by the absence of anion-specific transporters, the relative permselectivity ratio between K^+ and Na^+ can be estimated as:

$$\frac{p_{\mathrm{K}}}{p_{\mathrm{Na}}} = e^{\frac{V_{\mathrm{m}}F}{RT}} * \frac{[\mathrm{Na}^+]_{\mathrm{PEG}}}{[\mathrm{K}^+]_{\mathrm{DEX}}} \quad \text{(SE18)}$$

After membrane formation, the device behaves like a battery with open circuit voltage $V_{\mathrm{OC}} = V_{\mathrm{m}}$ and maximum power output P_{max}. These two quantities are related by[30,31]

$$P_{\mathrm{max}} = \frac{V_{\mathrm{OC}}^2}{4R_{\mathrm{int}}}, \quad \text{(SE19)}$$

where R_{int} is the internal resistance of the device. R_{int} can be estimated by measuring the voltage V_{L} across a resistor with resistance R_{L} connected with the ATPS to form a voltage divider (**Supplementary Figure 28**). A circuit was designed which enables to measure the V_{OC} and V_{L} when connected to a Keithley 2450 source meter (**Supplementary Figure 28**). Ag/AgCl electrodes with offset potential smaller than 1 mV were used during the measurement. R_{int} can be calculated from V_{OC} and V_{L} as:[30]

$$R_{\mathrm{int}} = R_{\mathrm{L}}\left(\frac{V_{\mathrm{OC}}}{V_{\mathrm{L}}} - 1\right). \quad \text{(SE20)}$$

Furthermore, the source meter was used to measure the short-circuit current, with no load (0 Ω) connected to the cell (**Supplementary Figure 28 B**).

Voltage output over time

The voltage output of a single ATPS-supported membrane with a gradient of 1 M KCl / 0.78 M NaCl, doped with 5 mol% VM, was measured for 40 minutes (**Supplementary Figure 29**). Simultaneously, using the circuit described in **Supplementary Figure 28**, the voltage under load (R = 4.6 MΩ) was measured. An above-average initial voltage of over 140 mV quite slowly dropped to around 100 mV in these 40 min, while the voltage under load started around 95 mV and gradually decreased to just below 70 mV. The calculated internal resistance was R_{int} = 2.7 MΩ, and derived maximum power was $P_{\mathrm{max}} = 2 \cdot 10^{-3}$ mW.

C. Supplementary Tables and Figures

Supplementary Table 1. Properties of BCP membranes formed in cylindrical cells with a cross-sectional diameter of 4.5 mm using PHMA and PDMS hydrophobic blocks.

**Property**	**PHMA-based membrane (n=3)**	**PDMS-based membrane (n=6)**
R_{SP} / MΩ*cm^2	0.2 – 0.35	0.4 – 0.7
C_{SP} / μF*cm^{-2}	0.05 – 0.20	0.1 – 0.30
d / nm	48 ± 23	33 ± 13

Supplemetary Table 2. Integrated fluorescence intensities and intensity ratios of: **Cy3 BCP**: only Cy3-labelled membrane with no Cy5; **Cy5 DEX**: only C5 in the DEX phase; **Cy5 PEO**: only Cy5 in the PEO phase; **Cy3 BCP+Cy5 DEX**: Cy3-labelled membrane formed with a DEX solution containing Cy5; **Cy3 BCP+Cy5 PEO**: Cy3-labelled membrane formed with a PEO solution containing Cy5.

	**Cy3 BCP**	**Cy5 DEX**	**Cy5 PEO**	**Cy3 BCP+Cy5 DEX**	**Cy3 BCP+Cy5 PEO**
$\boldsymbol{I}_{\mathbf{F}}^{\mathbf{Ch1}}$ (655-725 nm)	739	1421	1449	3130	4000
$\boldsymbol{I}_{\mathbf{F}}^{\mathbf{Ch2}}$ (525-575 nm)	4656	35	35	6710	4382
$\boldsymbol{R}_{\mathbf{FRET}}$	0,16	40,6	41,4	0,46	0,91

Supplemetary Table 3. Mean fluorescence lifetimes for **Cy3 BCP**: only Cy3-labelled membrane with no Cy5; **Cy5 DEX**: only C5 in the DEX phase; **Cy5 PEO**: only Cy5 in the PEO phase; **Cy3 BCP+Cy5 DEX**: Cy3-labelled membrane formed with a DEX solution containing Cy5; **Cy3 BCP+Cy5 PEO**: Cy3-labelled membrane formed with a PEO solution containing Cy5.

**Lifetime (ns)**	**Cy3 BCP**	**Cy5 DEX**	**Cy5 PEO**	**Cy3 BCP+Cy5 DEX**	**Cy3 BCP+Cy5 PEO**
$\boldsymbol{\tau}_{\mathbf{m}}^{\mathbf{Ch1}}$ (655-725 nm)	-	0.72	0.79	0.99	1.05
$\boldsymbol{\tau}_{\mathbf{m}}^{\mathbf{Ch2}}$ (525-575 nm)	0.74	-	-	0.74	0.70

Supplementary Table 4. Bonded interaction parameters, as defined in Equations SE11 and SE12, used in the CGMD simulations for all polymer/solvent species. Note the DMAEMA species has identical bonded interaction parameters to DEX for simplicity.

**Species**	Chain Length (CG Beads)	k_{bond} $(k_B T/\sigma)$	l_0 (σ)	k_{angle} $(k_B T/rad^2)$	θ_0
**DEX**	200	1111	0.967	1.0	180
**PEO**	200	1111	0.967	0.5	180
**DEX** (Head) (DMAEMA)	7	1111	0.967	1.0	180
**PEO** (Head)	7	1111	0.967	0.5	180
**DMS** (Tail)	16	1111	0.967		
**H_2O**					

Supplementary Table 5. Interaction parameters, as defined in Equation SE13, used in CGMD simulations describing the energetic attraction between polymer and solvent species.

ε_{AB}	DEX	PEO	DMS	W
DEX	1.0	0.9	0.4	0.8
PEO		1.0	0.4	0.8
DMS			0.5	0.1
W				0.5

Supplementary Table 6. List of the interaction parameters used for the SCF computations.

χ_{ij}	**Value**	**Motivation**
χ_{AB}	0.5, 1	$\chi_{AB} = 0.5 \rightarrow$ Weak repulsion, aqueous solutions of short A and B oligomers would mix while long polymers would demix. Used for most of the computations.<br><br>$\chi_{AB} = 1 \rightarrow$ The polymers are highly incompatible. Used to verify if strong PEO-DEX incompatibility drives the formation of asymmetric membranes
χ_{AD}	0-0.5	Varied to study how of this parameter affect the structure of the bilayer
χ_{AC}	0.5	The hydrophobic blocks tend to segregate from the hydrophilic blocks
χ_{AS}	0.45	Compatible with the experimental values for PEG in water
χ_{BC}	0.5	B (DEX) does not like to interact with hydrophobic blocks, but the repulsion is not strong
χ_{BD}	0	Simulates hard-core interactions making the D (PDMEMA or similar) blocks compatible with the B (DEX) phase, although no attraction is present
χ_{BS}	0.45	Compatible with the experimental values for DEX in water
χ_{CD}	0.5	The hydrophobic blocks tend to segregate from the hydrophilic blocks
χ_{CS}	2.5	Simulates strongly hydrophobic blocks such as PDMS
χ_{DS}	0.45	Similar to the ones of DEX and PEO in water

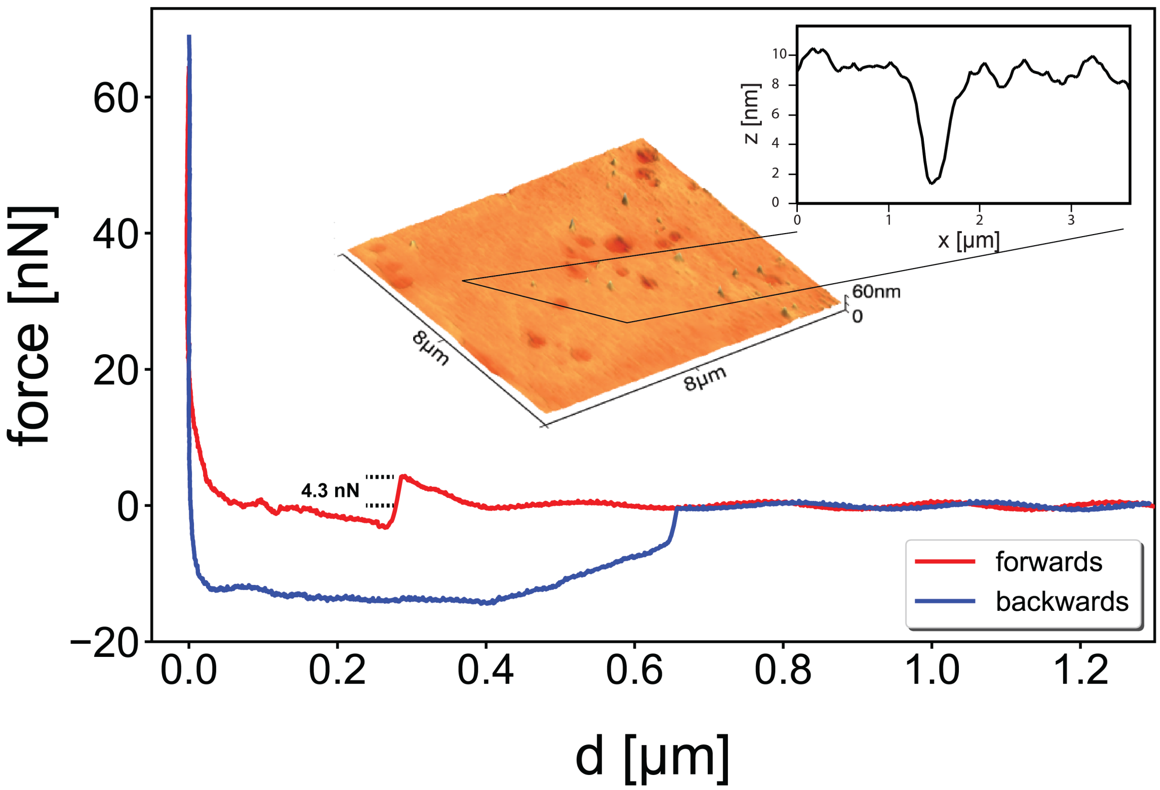

Supplementary Figure 1. AFM force vs. tip-sample distance plot from an AFM force-spectroscopy experiment. Around the distance of 0.3 µm, the forward curve shows the characteristic signature of a cantilever penetrating a membrane on a fluid support[32]. The backwards curve depicts the hysteresis caused by non-conservative force interactions between the membrane and the AFM cantilever tip. Insert: AFM micrograph of an ATPS-supported membrane in its hydrated state. The darker regions are variations in the membrane thickness of approximately 7 nm, as shown by the line profile in the top insert.

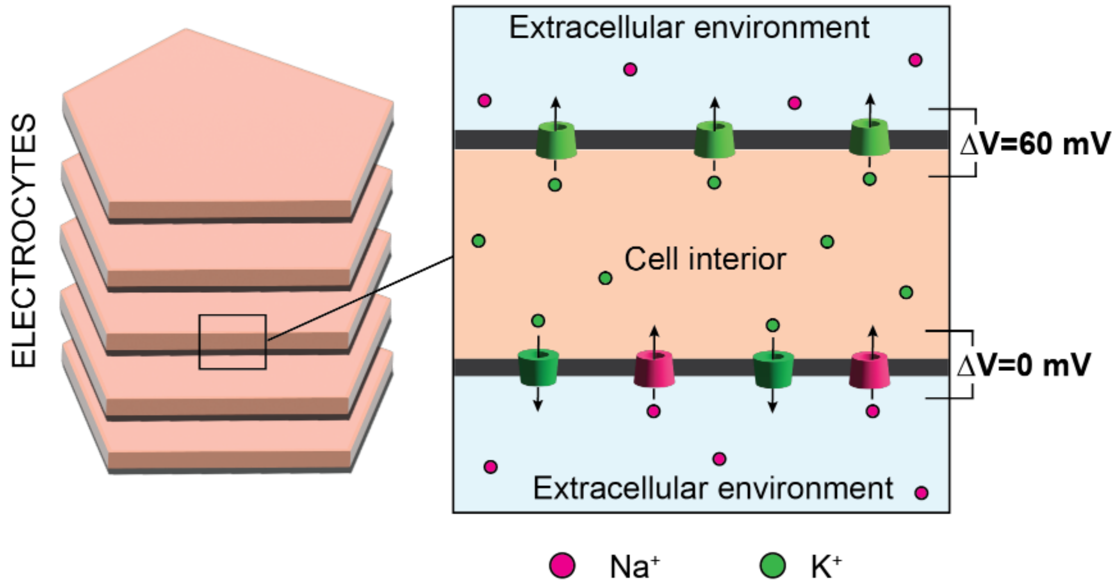

Supplementary Figure 2. Arrangement of the electrocytes and their electrochemical functioning in torpedo ray's electric organ; green and magenta channels represent K^+ and Na^+ ion selective channels, respectively.

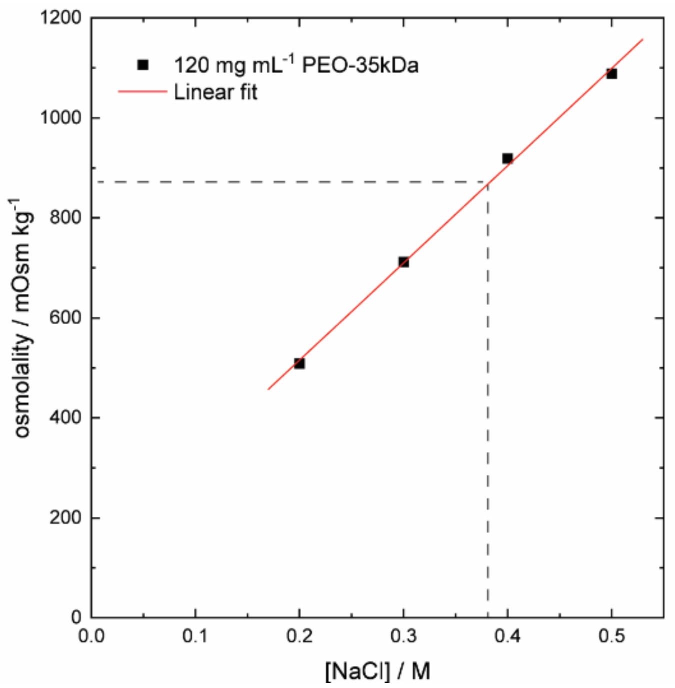

Supplementary Figure 3. Osmolality versus NaCl concentration for 120 mg mL^{-1} PEO (M_n = 35 kDa) solution. The red line is the result of a linear regression (R^2 = 0.99812) and the dashed lines indicate the 885 mOsm kg^{-1} (of the DEX solution in 0.5 M KCl) and the 0.39 M NaCl required to match that value.

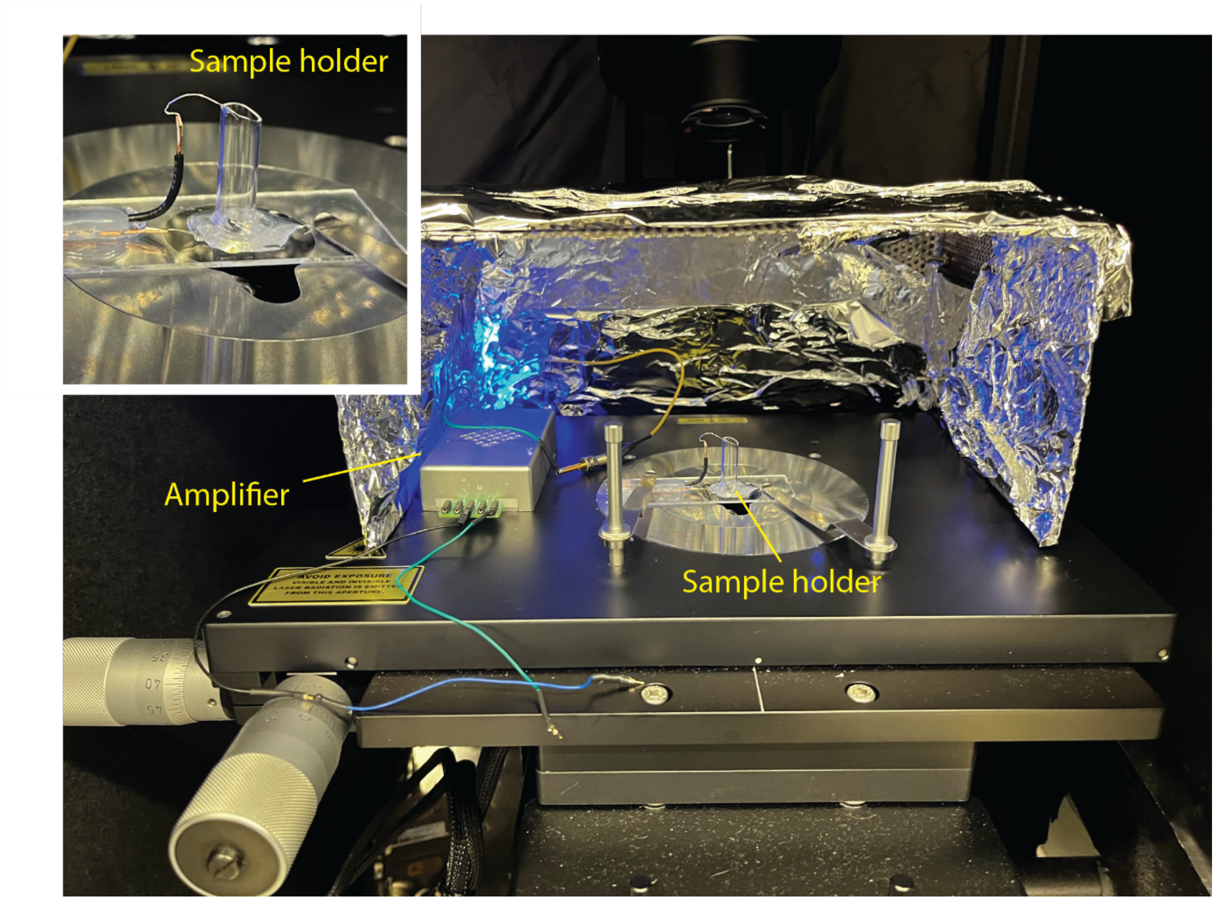

Supplementary Figure 4. Setup used for the FRET analysis of the ATPS-supported membranes

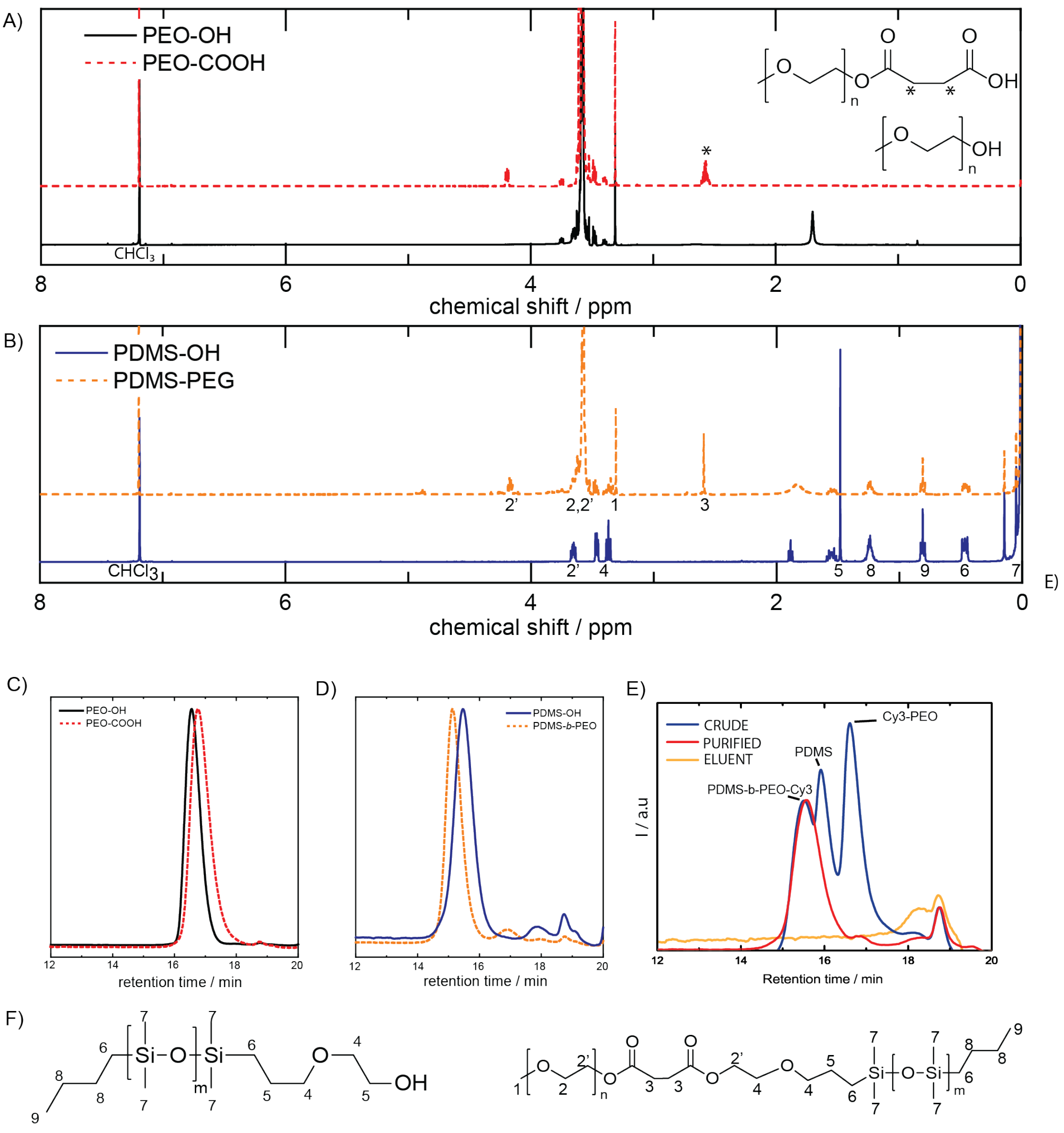

Supplementary Figure 5. ^{1}H-NMR spectra of A) PEO before (-OH) and after (-COOH) modification with succinic anhydride and B) of PDMS-OH and PDMS-*b*-PEO. Size-exclusion chromatograms of C) PEO before/after modification and D) of PDMS homopolymer and PDMS-*b*-PEO block copolymer. E) Size-exclusion chromatograms of PDMS-*b*-PEO-Cy3 before and after purification. F) Chemical structures of PDMS-OH and PDMS-*b*-PEO with different protons numbered corresponding to the respective chemical shifts in panel B.

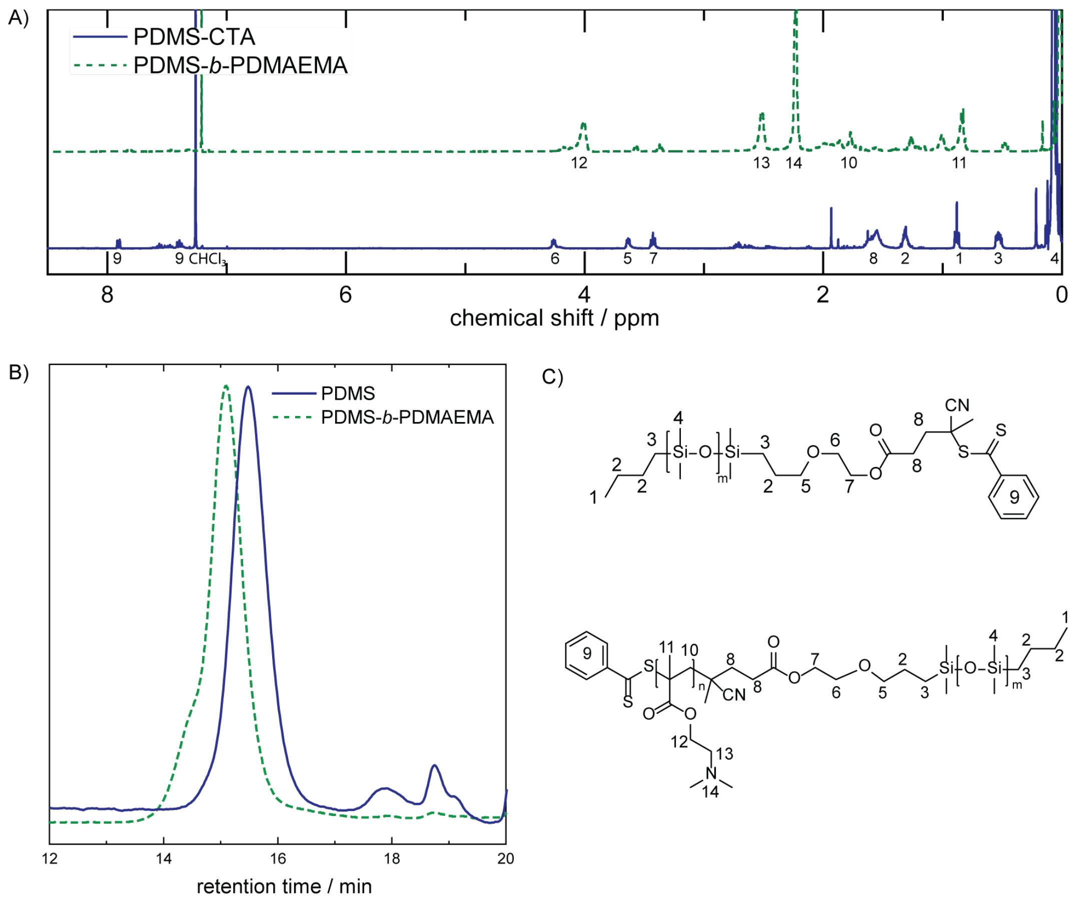

Supplementary Figure 6. A) ^{1}H-NMR spectra and B) size-exclusion chromatograms of PDMS-CTA and PDMS-*b*-PDMAEMA. C) Chemical structures of PDMS-CTA and PDMS-*b*-PDMAEMA with different protons numbered corresponding to the respective chemical shifts in panel A.

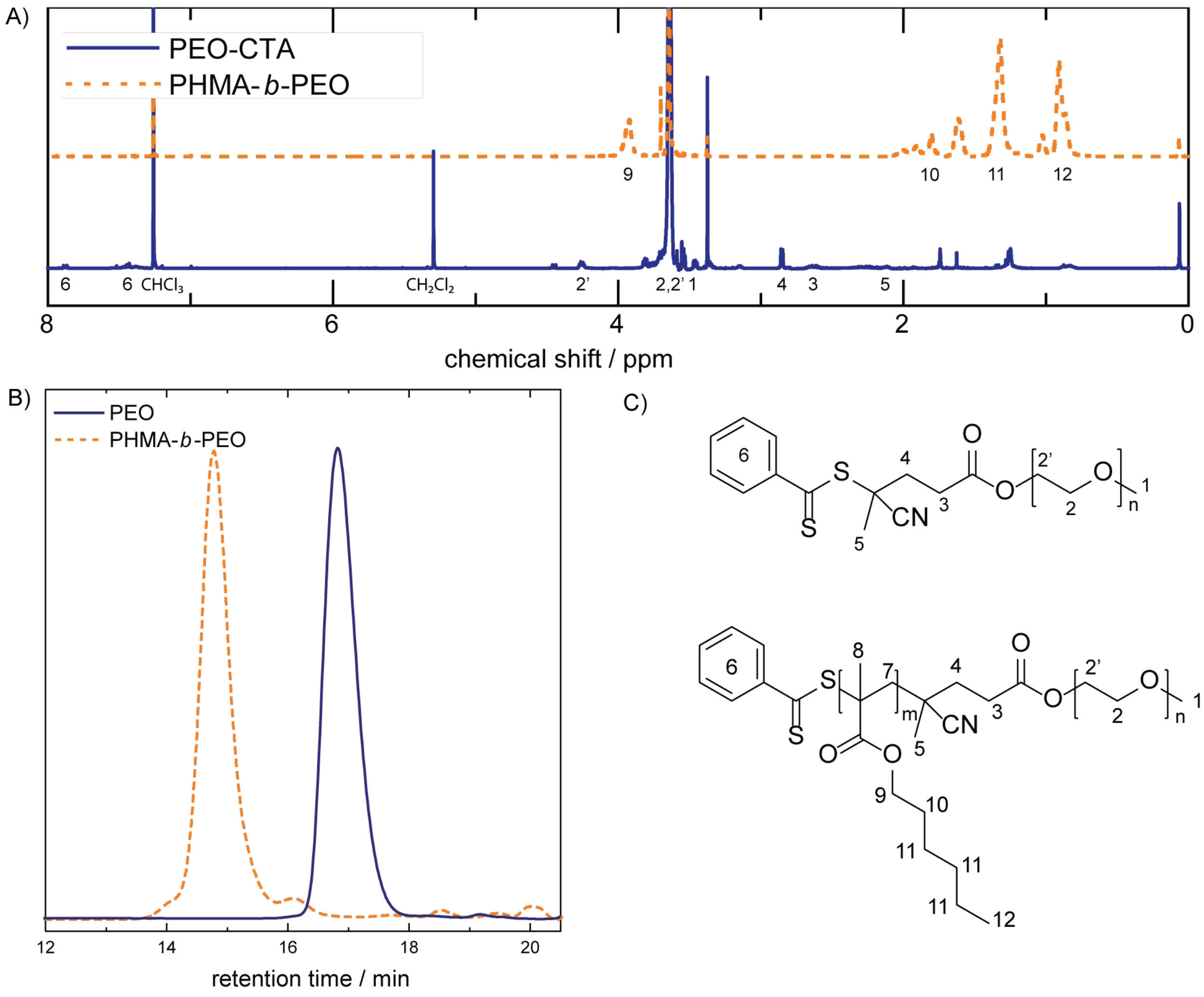

Supplementary Figure 7. A) ^{1}H-NMR spectra and B) size-exclusion chromatograms of PEO-CTA and PHMA-*b*-PEO. C) Chemical structures of PEO-CTA and PHMA-*b*-PEO with different protons numbered corresponding to the respective chemical shifts in panel A.

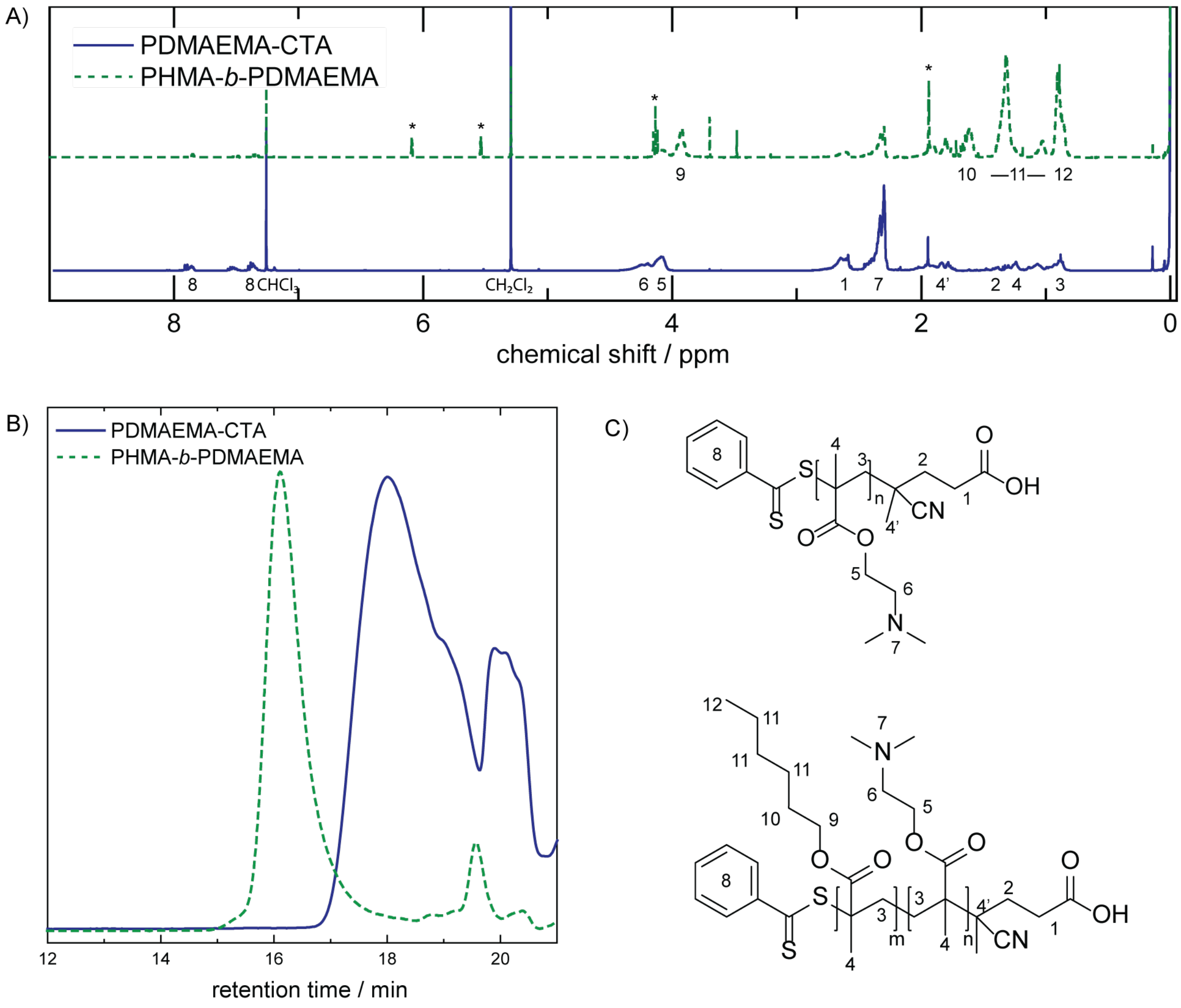

Supplementary Figure 8. A) ^{1}H-NMR spectra (* indicate peaks associated with unreacted HMA monomer) and B) size-exclusion chromatograms of PDMAEMA-CTA and PHMA-*b*-PDMAEMA. C) Chemical structures of PDMAEMA-CTA and PHMA-*b*-PDMAEMA with different protons numbered corresponding to the respective chemical shifts in panel A.

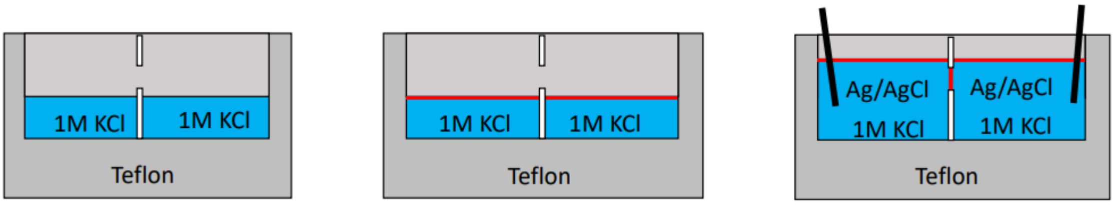

Supplementary Figure 9. Cross-sections of the Teflon chambers separated by a thin partition with a hole that were used in the bilayer folding method to form BCP membranes. From left to right: first, filling with salt solution reaching below the hole; then, pipetting BCP solution in toluene on top; lastly, raising the liquid level and folding a bilayer membrane across the hole.

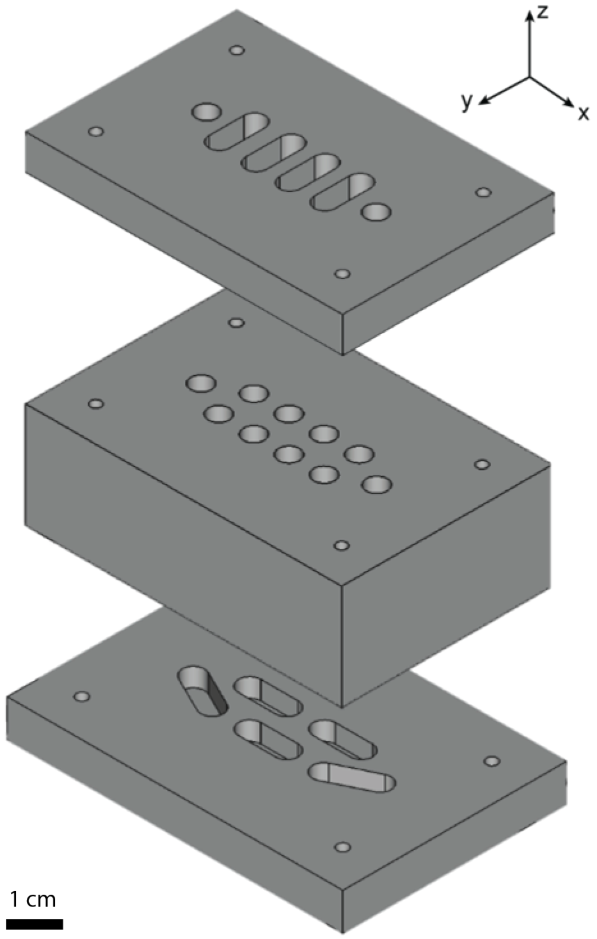

Supplementary Figure 10. 3D CAD model of the multicell electronic device consisting of three parts: the bottom connecting liquid columns in the x direction, the middle shaping the columns where the ATPS with membrane is formed and the top connecting the columns in the y direction.

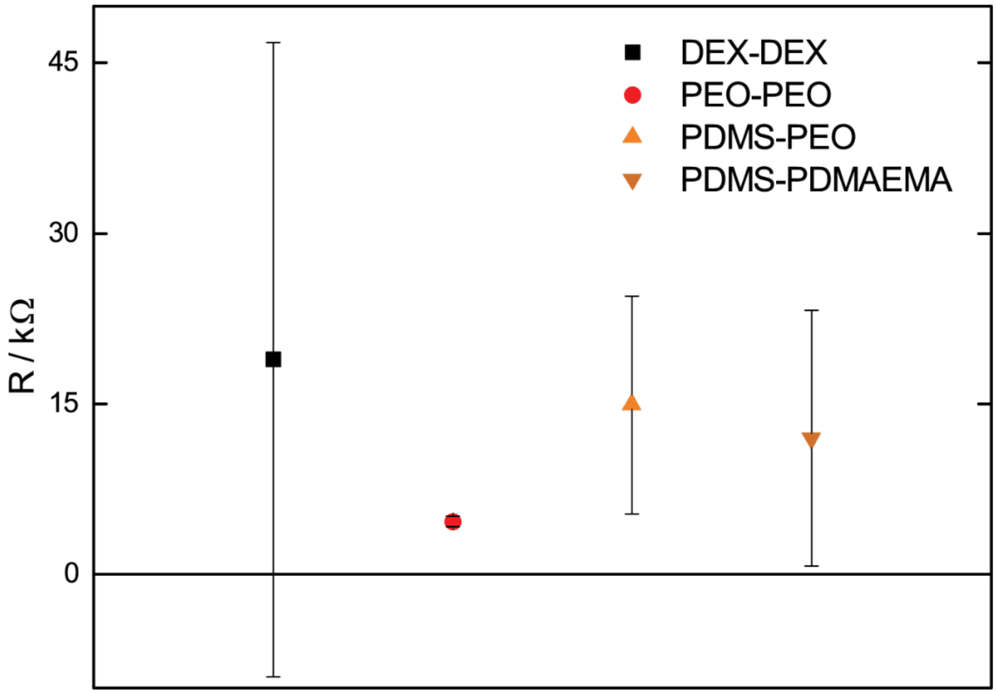

Supplementary Figure 11. Resistance measurement of the control experiments showing the absence of a membrane at ATPS interface.

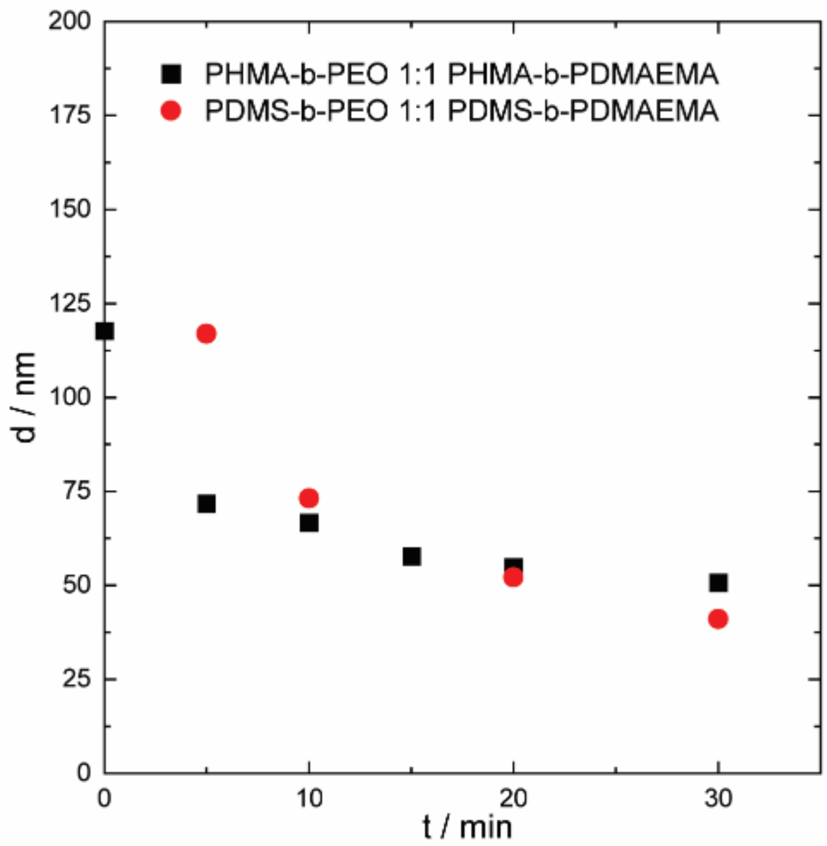

Supplementary Figure 12. Thickness of membranes formed at the ATPS interface versus time after formation, comparing membranes consisting of PHMA- and PDMS-based block copolymers. BCP concentration: 50 mg·mL^{-1} in toluene. ATPS: DEX in 0.5 M KCl and PEO in 0.39 M NaCl.

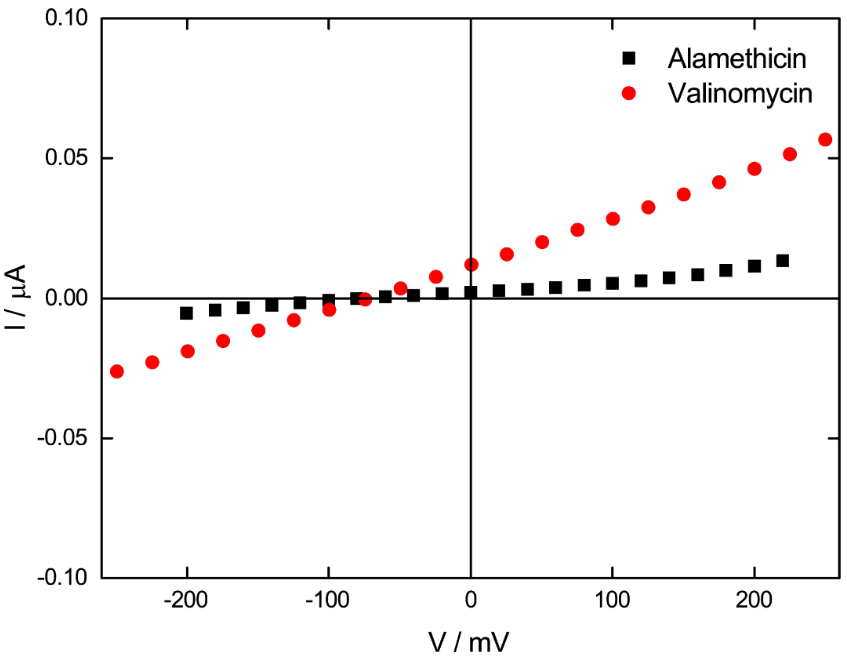

Supplementary Figure 13. Transmembrane current as a function of applied voltage comparing valinomycin and alamethicin (both at 2 mol% with respect to BCP). Membranes were formed with BCP1 and BCP2 using the planar bilayer folding method across a hole with 100 µm diameter. Salt concentrations in the two chambers of the setup were 1 M NaCl and 1 M KCl respectively.

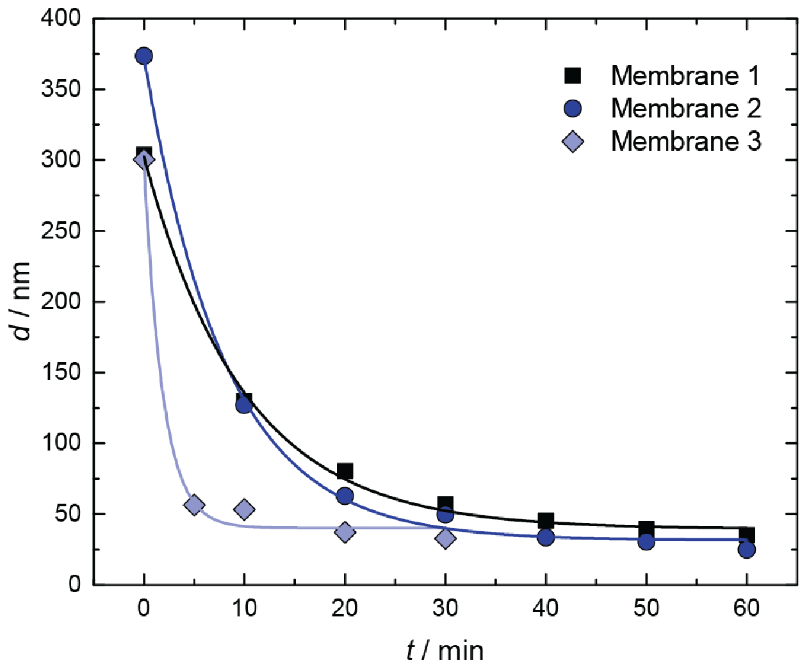

Supplementary Figure 14. Evolution of the thickness (d) of ATPS-stabilized BCP membranes prepared by the solvent displacement method as a function of equilibration time (t); the three membranes, made of BCP1 and BCP2, are repetitions of the same experiment.

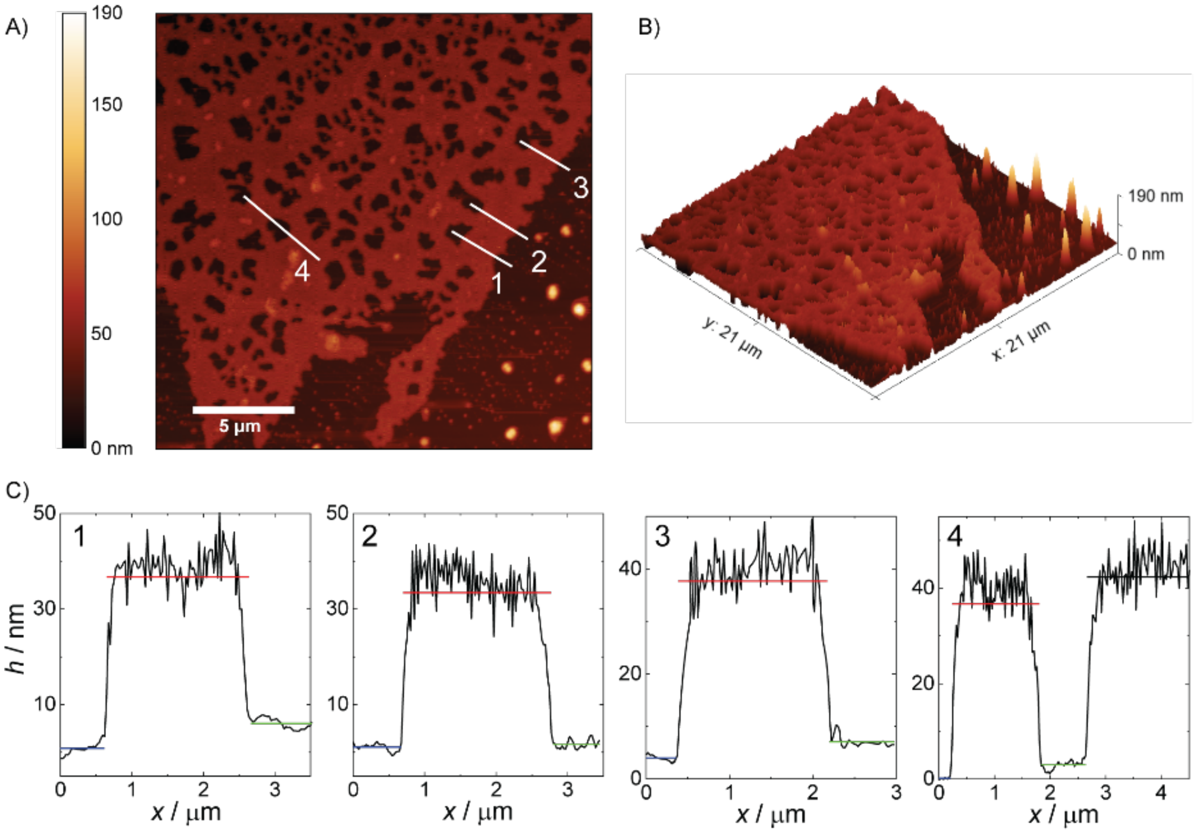

Supplementary Figure 15. Dry state atomic force microscopy (AFM) height image in A) 2D and B) 3D side view of a BCP (BCP1 + BCP2) membrane deposited from the ATPS interface onto a clean silicon wafer. Washing of the substrate was done very carefully with pure water to not remove the deposited membrane. The spikes on the right-hand side are assumed to be either clumps of remaining PEO or DEX from the ATPS or salt crystals. C) Line profiles as indicated in the first panel. Heights (approximately 35 nm) were calculated between the baseline (blue/green) and top average (red).

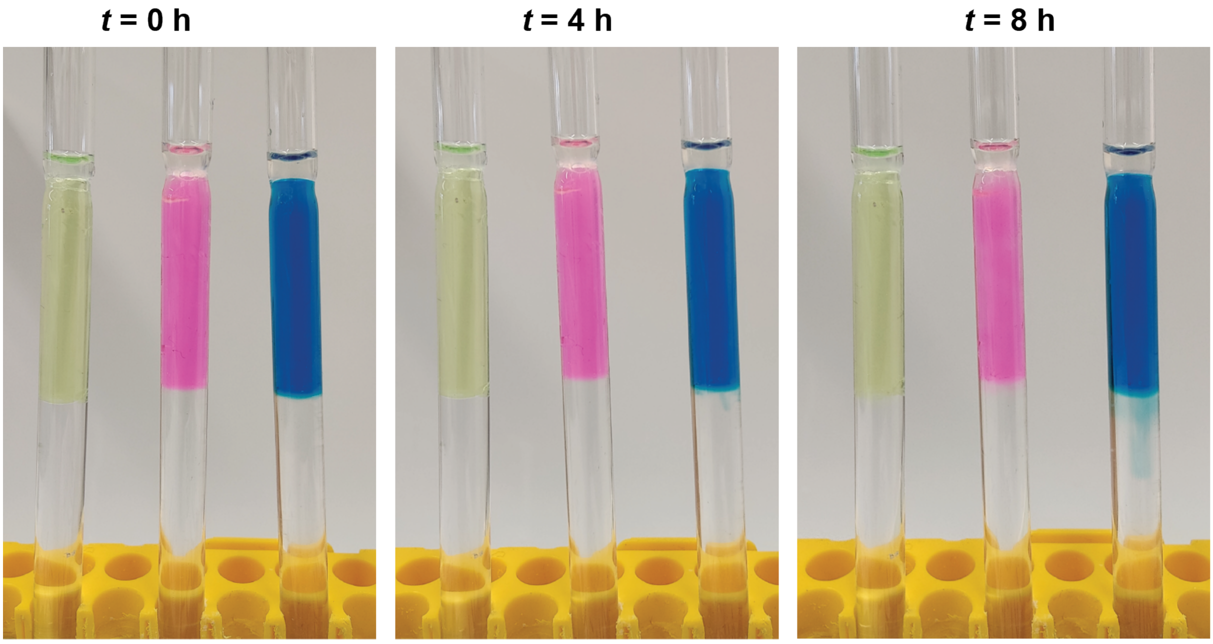

Supplementary Figure 16. Dye leakage test with - from left to right in each picture - calcein (anionic), rhodamine B (amphoteric), methylene blue (cationic) at three different times after the membranes were formed.

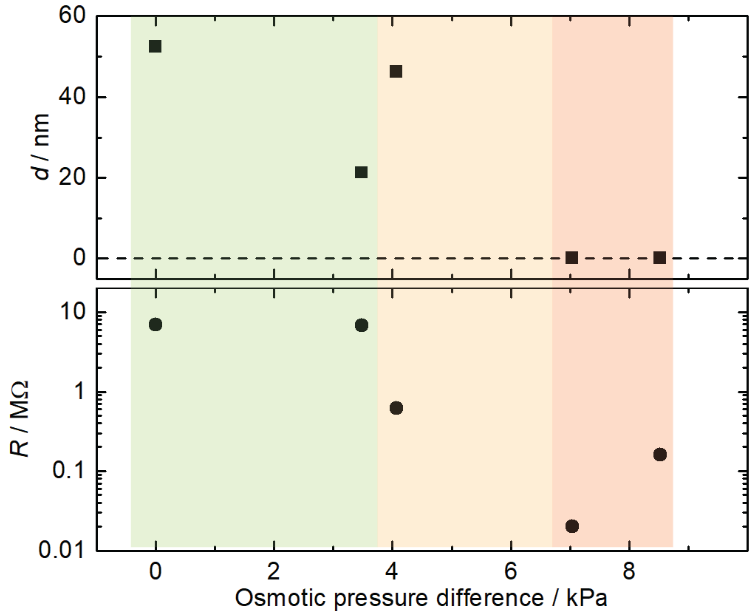

Supplementary Figure 17. Membrane formation using ATPS with various osmotic pressure differences, achieved via addition of NaCl in the PEO phase. The green region marks the stability range of the membranes. In the yellow region, membranes can be formed but their resistance is low, indicating that they possess defects. In the red region, no membrane formation was observed

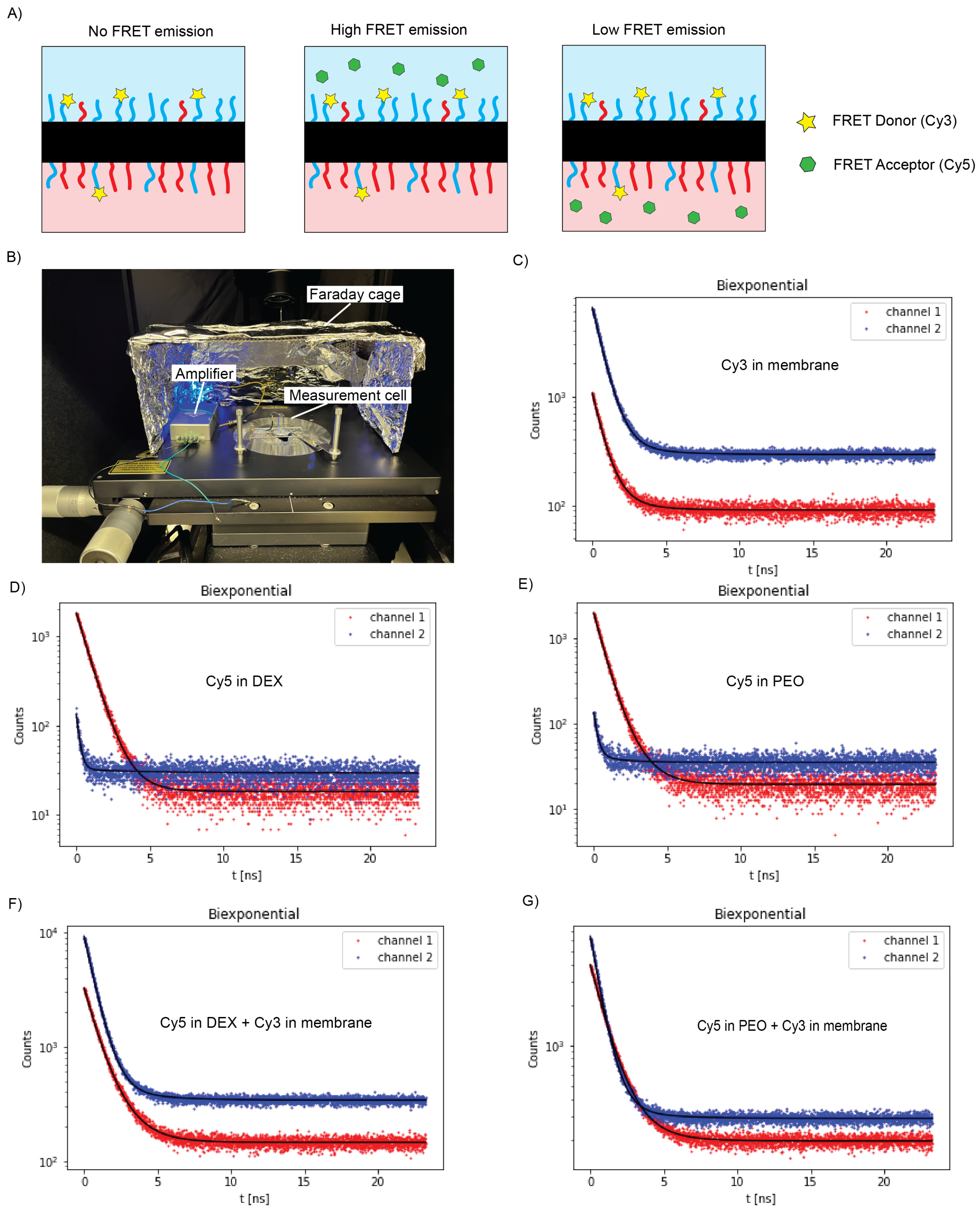

Supplementary Figure 18. Characterization of the ATPS-supported membranes by FRET. A) Schematic description of the FRET experiments performed to probe the asymmetry of the membrane. B) Custom-made microscopy setup employed for the FRET measurements. C-G) Time-Correlated Single Photon Counting histograms obtained from the C-F) control experiments and the F-G) FRET experiments. Details are provided in the panels. Channel 1 (655-725 nm) is the Cy5 channel while Channel 2 (532-575 nm) is the Cy3 channel.

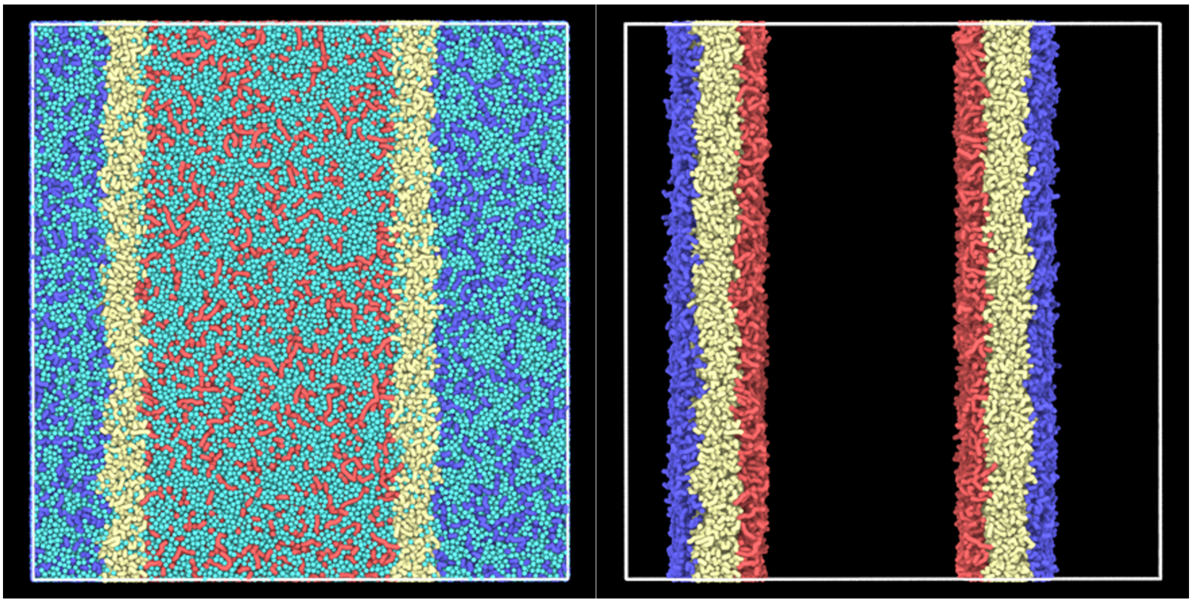

Supplementary Figure 19. Snapshots of the perfectly asymmetric bilayer, as used as a starting configuration prior to relaxation in CGMD simulations, at the interface between the two bulk-polymer phases. Solvent, DEX, PEO and PDMS beads are coloured in cyan, red, blue and yellow, respectively. All systems were visualised using OVITO[33]. The left panel shows all polymer/solvent species and the right panel shows only the BCPs with the aqueous polymer phases removed.

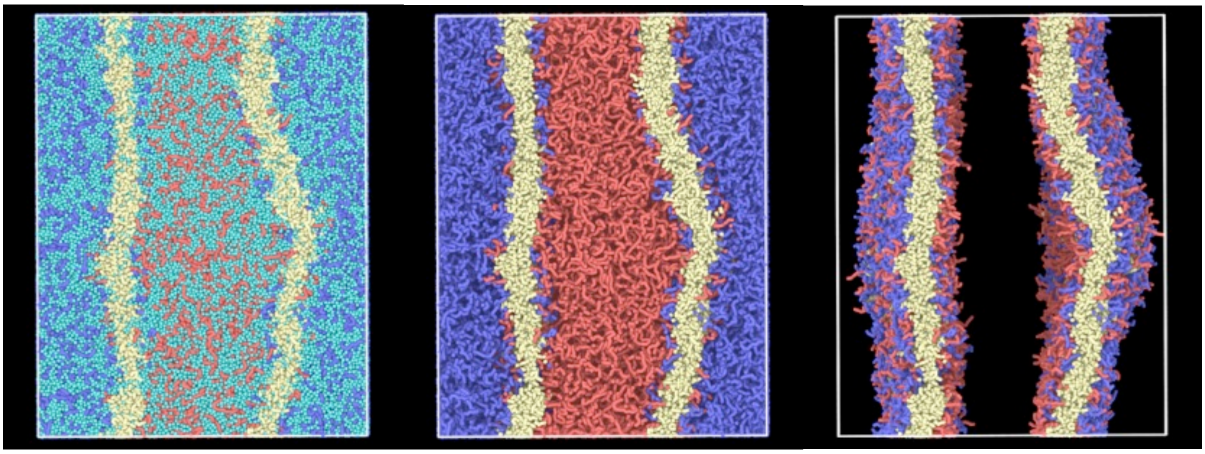

Supplementary Figure 20. Snapshots of the double bilayer system used in CGMD simulations after the equilibration procedure. (left) Full double bilayer structure with all components, (center) with only polymers and (right) with only BCPs components shown. Solvent, DEX, PEO and PDMS beads are coloured in cyan, red, blue and yellow respectively. Two bilayers are present because of the periodic boundary conditions. All systems were visualised using OVITO[33].

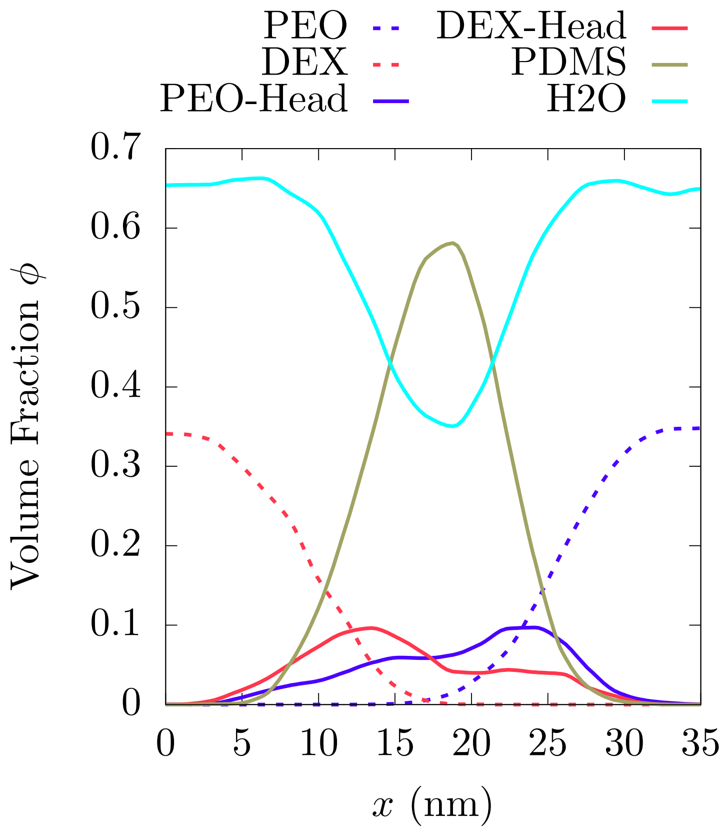

Supplementary Figure 21. Concentration profile, extracted from CGMD simulations, showing the volume fractions of the different components accumulated at the interface between the two aqueous phases. Blue and red dashed lines indicate the polymer phases, blue and red solid lines the PEO and DEX head groups of the BCPs, beige line the PDMS tails and the cyan line solvent beads. The profile shown here does not include the correction for undulations hence all peaks appear broader than the concentration profile shown in the main manuscript.

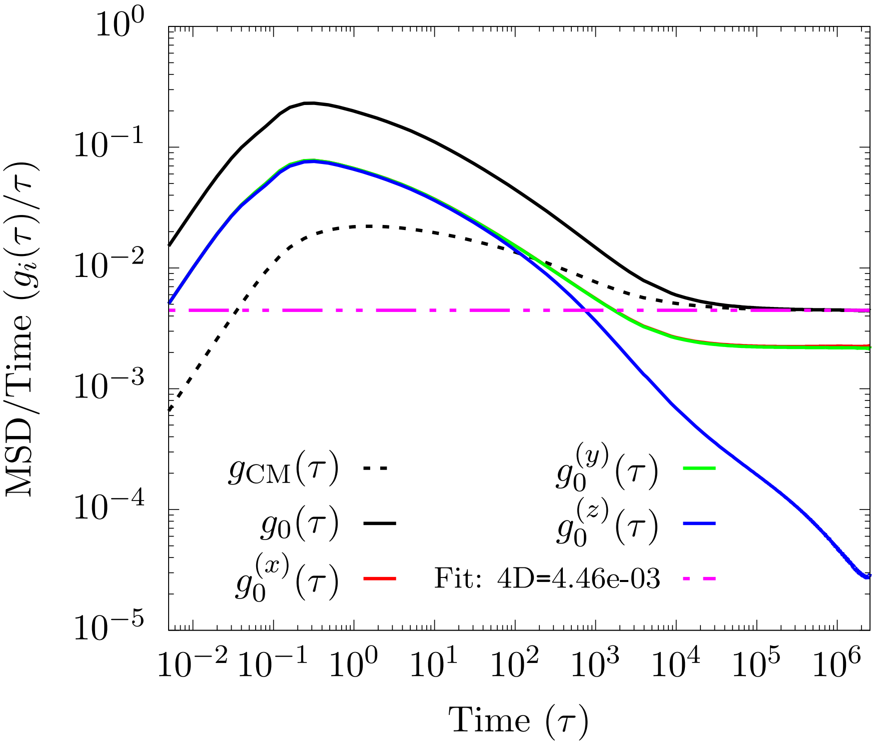

Supplementary Figure 22. Mean squared displacement of all monomers $g_0(\tau)$ and of the centre of mass $g_{CM}(\tau)$ of the BCP chains as a function of time extracted from CGMD simulations. The x, y and z components of the MSD are also shown, indicating quasi-2D diffusive behaviour in the x-y plane of the bilayer. The magenta line indicates a linear fit to extract the diffusion coefficient at long times and is stated in units of σ^2/τ.

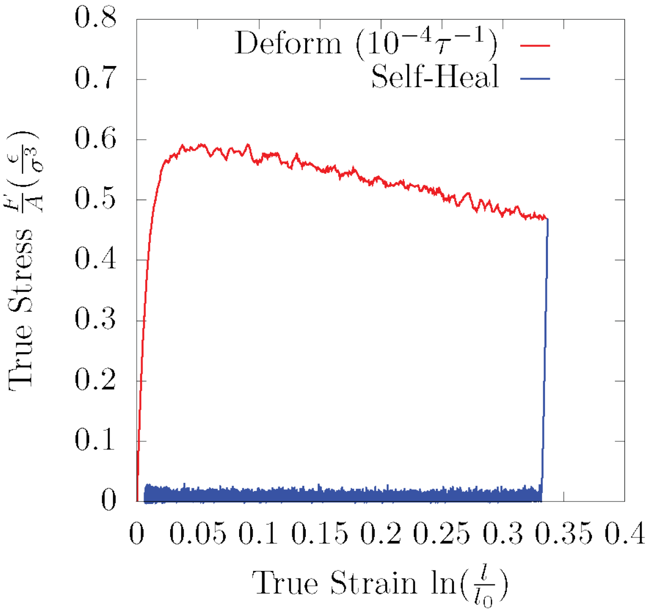

Supplementary Figure 23. Stress-strain curves during the deformation and self-healing stages of the autonomous self-healing CGMD simulations.

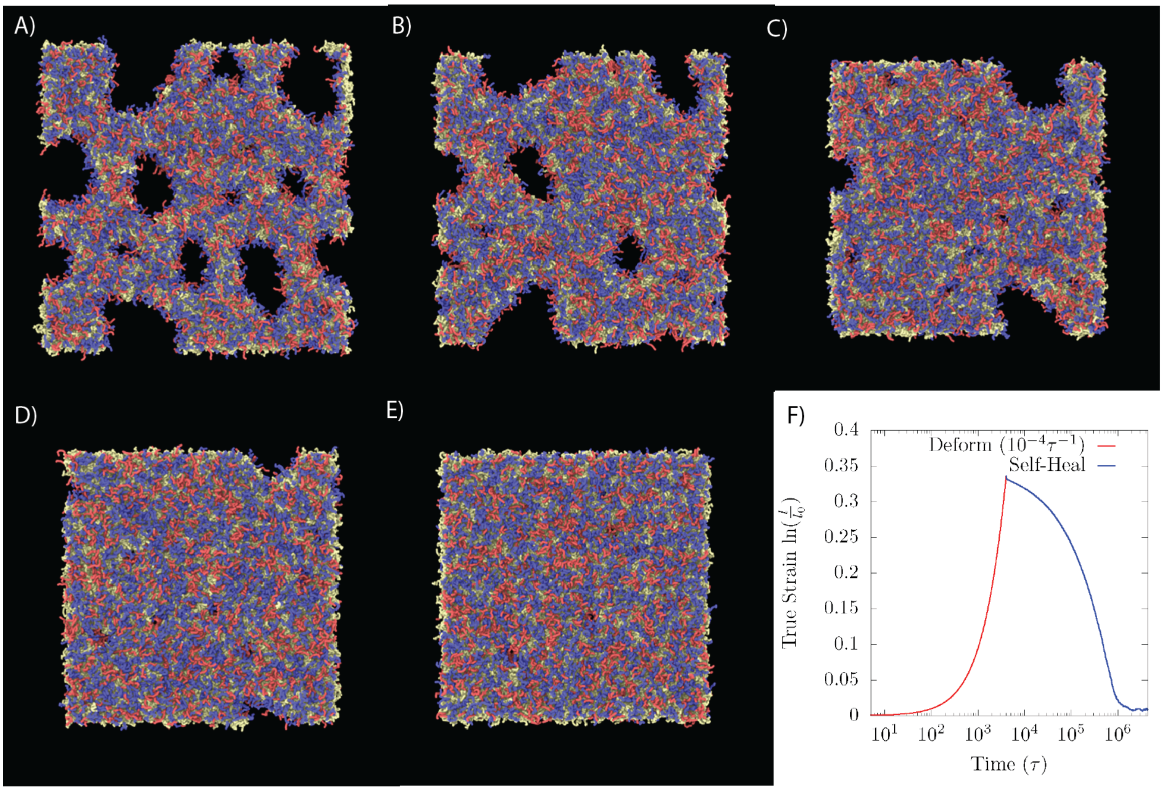

Supplementary Figure 24. System snapshots of the PEO face of the ATPS bilayer, as stabilized in CGMD simulations, during non-equilibrium deformation and self-healing simulations. A-E) A series of snapshots of the PEO side of the bilayer at different post deformation times, during self-healing (250kτ, 500kτ, 750$k\tau$, 1000$k\tau$, 1250$k\tau$, 1500$k\tau$). F) True-strain vs. time during deformation and self-healing.

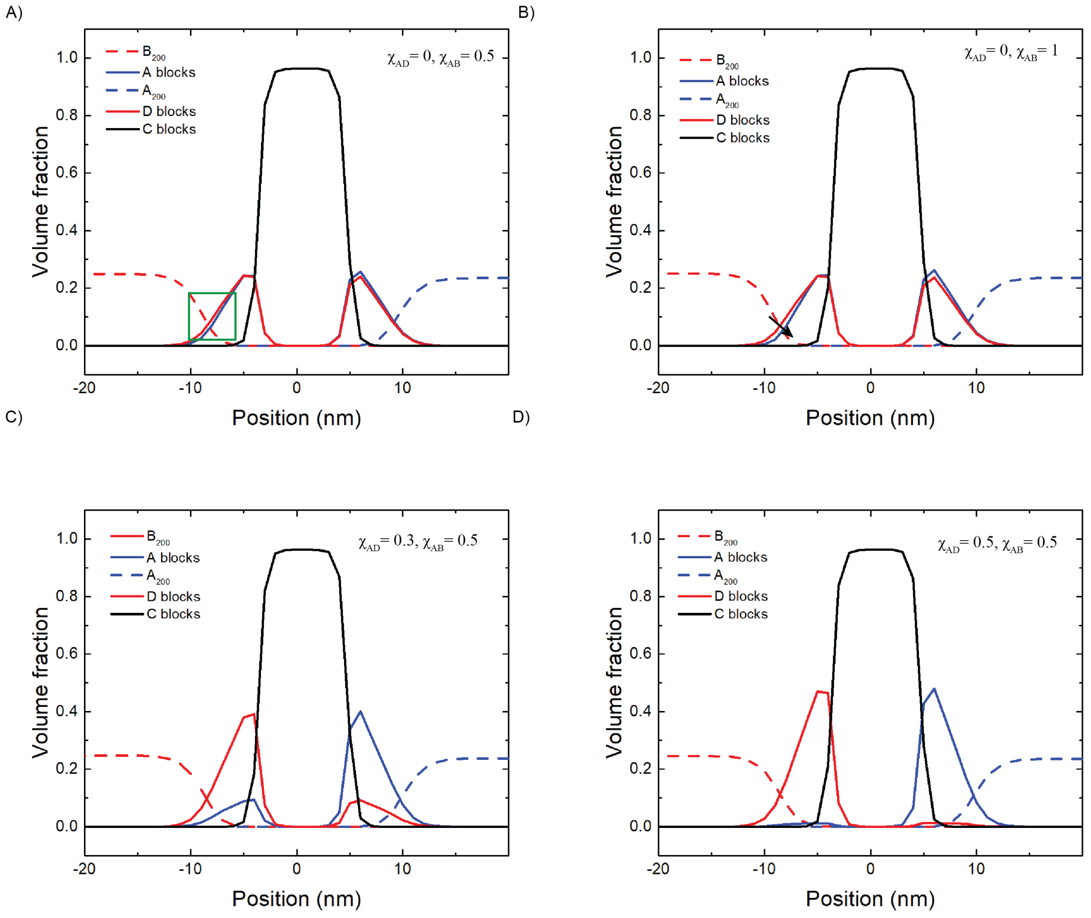

Supplementary Figure 25. Self-consistent field concentration profiles obtained with A_{20}-C_{40} and D_{20}-C_{40} block copolymers. The green box in panel A highlights the small asymmetry in the distribution of the blocks. The black arrow in panel B indicates an increase in the asymmetry of the distribution of the blocks. The interaction parameters used are specified in the graphs and in Supplementary Table 2.

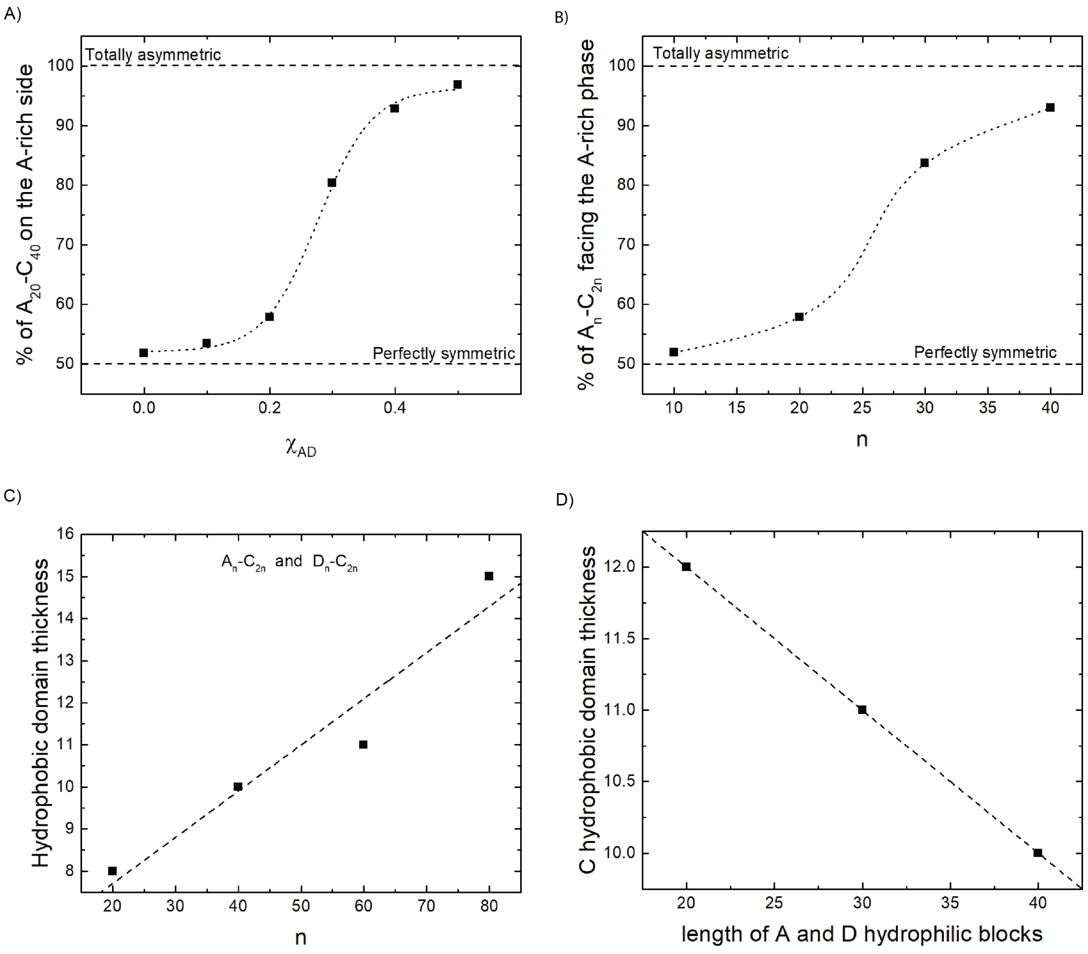

Supplementary Figure 26. Self-consistent field results. A, B) Distribution of the A_n-C_{2n} block copolymers in the bilayer as a function of A) the interaction parameter χ_{AD} and B) the block length parameter n. C) Thickness of the hydrophobic domain of the bilayer as a function of the block length parameter n for block copolymers of composition A_n-C_{2n} and D_n-C_{2n}. D) Thickness of the hydrophobic domain of the bilayer as a function of the length of the hydrophilic blocks at a constant hydrophilic block length (C_{60}). The parameters used in the calculations are the ones specified in Supplementary Table 2 with $\chi_{AD} = 0.5$ and $\chi_{AB} = 0.5$.

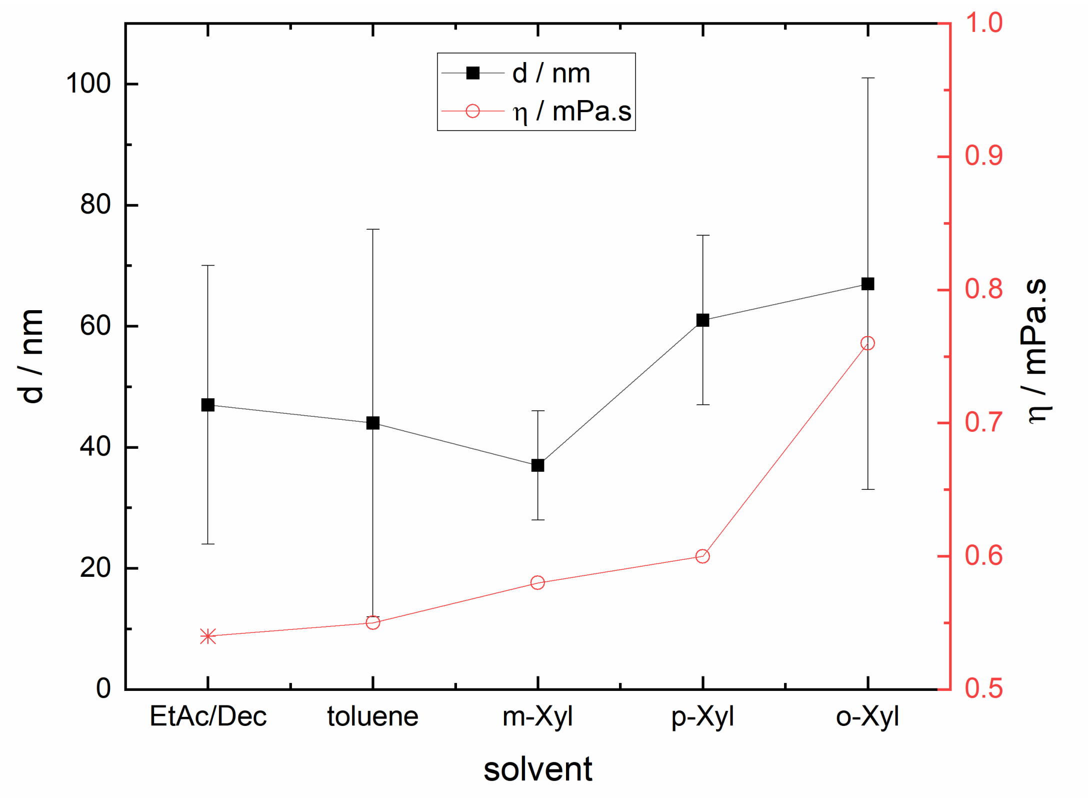

Supplementary Figure 27. Membrane bilayer thickness (black squares) when using various organic solvents for the BCP solution (50 mg mL^{-1}) and the dynamic viscosity of the solvents (red circles); error bars indicate standard deviation between different membranes. The EtAc/Dec mixture consists of 75% (v/v) ethyl acetate and 25% (v/v) n-decane. *viscosity was not measured but interpolated from the pure solvent viscosities. Each data point is the mean of 3 experiments

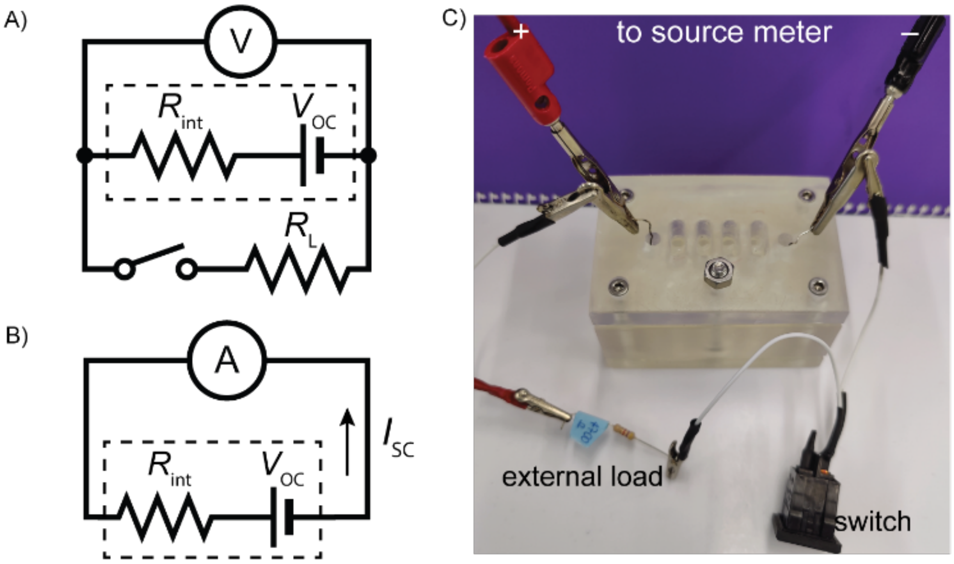

Supplementary Figure 28. Electrochemical setup. A) electric circuit to measure the V_{OC} and R_{int} of the cells. B) Electric circuit to measure the I_{SC} of the cells. C) Photo of the connected cells according to circuit A.

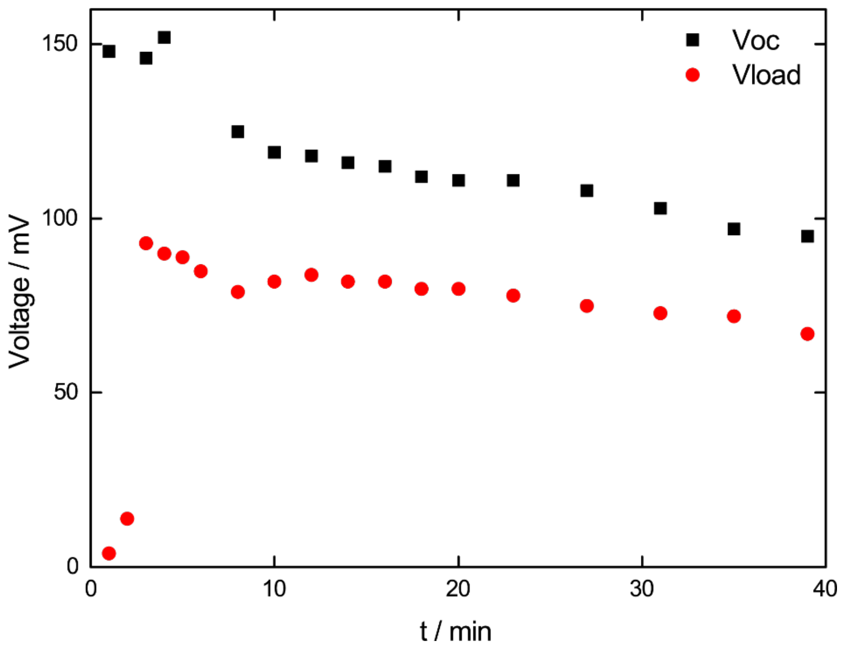

Supplementary Figure 29. Open-circuit voltage and voltage under load (R = 4.6 MΩ) measured over time for a single ATPS-supported, VM-doped (5%) bilayer membrane made of BCP1 and BCP2 with a gradient of 1.0 M KCl in DEX / 0.78 M NaCl in PEO.

D. Supplementary References

1. Montal, M. & Mueller, P. Formation of Bimolecular Membranes from Lipid Monolayers and a Study of Their Electrical Properties. *Proceedings of the National Academy of Sciences* **69**, 3561–3566 (1972).

2. Naumowicz, M., Petelska, A. D. & Figaszewski, Z. A. Capacitance and resistance of the bilayer lipid membrane formed of phosphatidylcholine and cholesterol. *Cell Mol Biol Lett* **8**, 5–18 (2003).

3. Kangül, M. Advancing atomic force microscopy through open source instrumentation. (EPFL, Lausanne, 2023).

4. Watanabe, T., Ohtsuka, A., Murase, N., Barth, P. & Gersonde, K. NMR studies on water and polymer diffusion in dextran gels. Influence of potassium ions on microstructure formation and gelation mechanism. *Magn Reson Med* **35**, (1996).

5. Chen, L., Shen, H. & Eisenberg, A. Kinetics and Mechanism of the Rod-to-Vesicle Transition of Block Copolymer Aggregates in Dilute Solution. *J Phys Chem B* **103**, 9488–9497 (1999).

6. Zhulina, E. B. & Borisov, O. V. Theory of block polymer micelles: Recent advances and current challenges. *Macromolecules* vol. 45 4429–4440 Preprint at https://doi.org/10.1021/ma300195n (2012).

7. Hanke, W. & Schlue, W.-R. *Planar Lipid Bilayers*. (Elsevier, 1993). doi:10.1016/C2009-0-03331-5.

8. Montal, M. & Mueller, P. Formation of Bimolecular Membranes from Lipid Monolayers and a Study of Their Electrical Properties. *Proceedings of the National Academy of Sciences* **69**, 3561–3566 (1972).

9. Winterhalter, M. Black lipid membranes. *Curr Opin Colloid Interface Sci* **5**, 250–255 (2000).

10. Naumowicz, M., Petelska, A. D. & Figaszewski, Z. A. Capacitance and resistance of the bilayer lipid membrane formed of phosphatidylcholine and cholesterol. *Cell Mol Biol Lett* **8**, 5–18 (2003).

11. Qian, S., Wang, C., Yang, L. & Huang, H. W. Structure of the alamethicin pore reconstructed by x-ray diffraction analysis. *Biophys J* **94**, (2008).

12. Nardin, C., Winterhalter, M. & Meier, W. Giant Free-Standing ABA Triblock Copolymer Membranes. *Langmuir* **16**, 7708–7712 (2000).

13. Pietraszewska-Bogiel, A. & Gadella, T. W. J. FRET microscopy: From principle to routine technology in cell biology. *J Microsc* **241**, (2011).

14. Long, D. & Lequeux, F. Heterogeneous dynamics at the glass transition in van der Waals liquids, in the bulk and in thin films. *European Physical Journal E* **4**, (2001).

15. Peter, S., Meyer, H. & Baschnagel, J. MD simulation of concentrated polymer solutions: Structural relaxation near the glass transition. *European Physical Journal E* **28**, (2009).

16. Mark, J. E. Physical Properties of Polymers Handbook 2nd Edition. in *Springer Science+Business Media, LLC* (2007).

17. Hansen, J. P. & McDonald, I. R. *Theory of Simple Liquids: With Applications to Soft Matter: Fourth Edition*. *Theory of Simple Liquids: With Applications to Soft Matter: Fourth Edition* (2013). doi:10.1016/C2010-0-66723-X.

18. Plimpton, S. Fast parallel algorithms for short-range molecular dynamics. *J Comput Phys* **117**, (1995).

19. Thompson, A. P. *et al.* LAMMPS - a flexible simulation tool for particle-based materials modeling at the atomic, meso, and continuum scales. *Comput Phys Commun* **271**, (2022).

20. Fleer, G. J., Cohen-Stuart, M. A., Scheutjens, J. M. H. M., Cosgrove, T. & Vincent, B. *Polymers at Interfaces*. (Springer, 1998).

21. Mocan, M., Kamperman, M. & Leermakers, F. A. M. Microphase segregation of diblock copolymers studied by the self-consistent field theory of Scheutjens and Fleer. *Polymers (Basel)* **10**, (2018).

22. Lyatskaya, Y. V, Leermakers, F. A. M., Fleer, G. J., Zhulina, E. B. & Birshtein, T. M. Analytical Self-Consistent-Field Model of Weak Polyacid Brushes. *Macromolecules* vol. 28 3562–3569 Preprint at https://pubs.acs.org/sharingguidelines (1995).

23. Flory, P. J. Thermodynamics of high polymer solutions. *The Journal of Chemical Physics* vol. 9 Preprint at https://doi.org/10.1063/1.1750971 (1941).

24. De Gennes, P. G. Scaling concepts in polymer physics. Cornell university press. *Ithaca N.Y.,* Preprint at (1979).

25. Lindvig, T., Michelsen, M. L. & Kontogeorgis, G. M. A Flory-Huggins model based on the Hansen solubility parameters. *Fluid Phase Equilib* **203**, (2002).

26. Ianiro, A. *et al.* Liquid–liquid phase separation during amphiphilic self-assembly. *Nat Chem* **11**, (2019).

27. Stefanis, E. & Panayiotou, C. Prediction of hansen solubility parameters with a new group-contribution method. *Int J Thermophys* **29**, (2008).

28. Vena, M. P., de Moor, D., Ianiro, A., Tuinier, R. & Patterson, J. P. Kinetic state diagrams for a highly asymmetric block copolymer assembled in solution. *Soft Matter* **17**, 1084–1090 (2021).

29. Bowman, C. L. & Baglioni, A. Application of the Goldman-Hodgkin-Katz current equation to membrane current-voltage data. *J Theor Biol* **108**, 1–29 (1984).

30. Makarov, S. N., Ludwig, R. & Bitar, S. J. *Practical Electrical Engineering*.

31. Schroeder, T. B. H. *et al.* An electric-eel-inspired soft power source from stacked hydrogels. *Nature* **552**, 214–218 (2017).

32. Penedo, M. *et al.* Visualizing intracellular nanostructures of living cells by nanoendoscopy-AFM. *Sci Adv* **7**, (2021).

33. Stukowski, A. Visualization and analysis of atomistic simulation data with OVITO-the Open Visualization Tool. *Model Simul Mat Sci Eng* **18**, (2010).
